# Developmental Flower and Rhizome Morphology in *Nuphar* (Nymphaeales): An Interplay of Chaos and Stability

**DOI:** 10.3389/fcell.2020.00303

**Published:** 2020-05-19

**Authors:** Elena S. El, Margarita V. Remizowa, Dmitry D. Sokoloff

**Affiliations:** ^1^Department of Higher Plants, Faculty of Biology, M.V. Lomonosov Moscow State University, Moscow, Russia; ^2^Faculty of Biology and Biotechnologies, National Research University Higher School of Economics, Moscow, Russia

**Keywords:** angiosperms, development, evolution, flower, gravitropism, mechanical forces, phyllotaxis

## Abstract

European species of *Nuphar* are amongthe most accessible members of the basal angiosperm grade, but detailed studies using scanning electron microscopy are lacking. We provide such data and discuss them in the evolutionary context. Dorsiventral monopodial rhizomes of *Nuphar* bear foliage leaves and non-axillary reproductive units (RUs) arranged in a Fibonacci spiral. The direction of the phyllotaxis spiral is established in seedlings apparently environmentally and maintained through all rhizome branching events. The RUs can be located on dorsal, ventral or lateral side of the rhizome. There is no seasonality in timing of their initiation. The RUs usually form pairs in positions N and N + 2 along the ontogenetic spiral. New rhizomes appear on lateral sides of the mother rhizome. A lateral rhizome is subtended by a foliage leaf (N) and is accompanied by a RU in the position N + 2. We hypothesize a two-step process of regulation of RU/branch initiation, with the second step possibly involving environmental factors such as gravitropism. Each RU has a short stalk, 1-2 scale-like phyllomes and a long-pedicellate flower. We support a theory that the flower is lateral to the RU axis. The five sepals initiate successively and form two whorls as 3 + 2. The sepal arrangement is not ‘intermediate’ between whorled and spiral. Mechanisms of phyllotaxis establishment differ between flowers and lateral rhizomes. Petal, stamen and carpel numbers are not precisely fixed. Petals are smaller than sepals and form a whorl. They appear first in the sectors of the outer whorl sepals. The stamen arrangement is whorled to chaotic. The merism of the androecium tends to be the same as in the corolla. Flowers with odd numbers of stamen orthostichies are found. These are interpreted as having a non-integer merism of the androecium (e.g., 14.5). Carpels form a whorl in *N. lutea* and normally alternate with inner whorl stamens. Sterile second whorl carpel(s) are found in some flowers of *N. pumila.*

## Introduction

The question of the origin and early evolution of angiosperms and angiosperm flowers remains one of key problems of evolutionary botany (Bateman et al., 2006; Doyle, 2008, 2012; Friis et al., 2011; Herendeen et al., 2017; Wang, 2018; Coiro et al., 2019; Bateman, 2020). Despite the fascinating progress during recent decades, inferring patterns of evolution of floral characters is in some cases problematic or the analyses provide equivocal results (Doyle and Endress, 2000; Endress and Doyle, 2009; Sauquet et al., 2017, 2018; De-Paula et al., 2018; Sokoloff et al., 2018a; Rümpler and Theißen, 2019). Among important limitations of ancestral character reconstructions is the lack of data or insufficient knowledge of morphological and especially developmental characters in many angiosperms species (Sauquet et al., 2017; Sauquet and Magallón, 2018; Sokoloff et al., 2018a).

Throughout the centuries of research in developmental plant morphology, European species *Nuphar* (Nymphaeaceae, Nymphaeales) have been among the most accessible plants currently recognized as members of the basal angiosperm grade. In spite of the great amount of relevant publications and controversial morphological interpretations (Trecul, 1845; Raciborski, 1894a, b; Cutter, 1957a, b, 1958, 1959, 1961; Dormer and Cutter, 1959; Chassat, 1962; Moseley, 1965, 1972; Wolf, 1991; Igersheim and Endress, 1998; Endress, 2001; Schneider et al., 2003; Padgett, 2007; Endress and Doyle, 2009) a comprehensive developmental study of European species of *Nuphar* using scanning electron microscopy is lacking. We are filling this gap and discuss the importance of *Nuphar* for understanding early evolution of angiosperms.

Traditionally, *Nuphar* was regarded as sister to the rest of Nymphaeaceae, a conclusion well-supported by several morphological characters, including superior rather than (semi)inferior ovary (Les et al., 1999; Borsch et al., 2008; Taylor, 2008; see also He et al., 2018). The traditional circumscription of Nymphaeaceae was supported by the occurrence of syncarpy and other characters (e.g., Borsch et al., 2008). Among two other families of the order, Cabombaceae possess free carpels whereas pistils of Hydatellaceae are unicarpellate (Moseley et al., 1984; Igersheim and Endress, 1998; Rudall et al., 2007; Sokoloff et al., 2013). Recent evidence from plastid phylogenetics suggests that placement of *Nuphar* as sister to Cabombaceae cannot be ruled out (Gruenstaeudl et al., 2017; Gruenstaeudl, 2019). As pointed out by Gruenstaeudl (2019), the monophyly of Nymphaeaceae currently remains indeterminate, and specific phylogenetic conclusions are strongly dependent on the precise plastome gene, data partitioning scheme, and codon position evaluated. Other potential problems may include taxon sampling and long-branch effects. The ambiguity in placement of *Nuphar* makes ancestral state reconstruction even more problematic for some characters (especially syncarpy). In this situation, detailed knowledge on morphology of *Nuphar* is important. The genus consists of the primarily Eurasian section *Nuphar* and the American section *Astylus* (Padgett, 2007). To our knowledge, developmental data documented by scanning electron microscopy are only available for two American species, *N. advena* (Endress, 2001) and *N. polysepala* (Schneider et al., 2003). Though extremely useful, published illustrations do not cover all stages of flower development.

The waterlilies possess a lot of interesting and unusual structural and developmental features whose interpretation is problematic. Disentangling these controversies is important for accurate assessment of morphological evolution. For example, lateral branching is normally axillary in seed plants, both in their vegetative parts and infloresceces (e.g., Gatsuk, 1974), but morphological interpretation of shoot branching and especially flower arrangement in all families of Nymphaeales is controversial (Raciborski, 1894a, b; Cutter, 1957a, b, 1958, 1959; Chassat, 1962; Richardson, 1969; Moseley, 1972; Schneider et al., 2003; Grob et al., 2006; Endress and Doyle, 2009; Sokoloff et al., 2009). Interpretation of flower position in *Nuphar* is especially problematic, because the flower of *Nuphar* is associated with a minute phyllome (or two phyllomes) variously interpreted as flower-subtending bract belonging to the rhizome, homolog of the first sepal of *Nymphaea* or phyllome of the lateral axis (Trecul, 1845; Raciborski, 1894a; Cutter, 1959; Chassat, 1962; Moseley, 1972; Endress and Doyle, 2009). Another important question is interpretation of perianth and androecium phyllotaxis of *Nuphar* as whorled or spiral (Hiepko, 1965; Cronquist, 1981; Wolf, 1991; Endress, 2001; Schneider et al., 2003; Padgett, 2007). This is related to the question of whorled vs. spiral arrangement of floral parts in ancestral flowers (Sauquet et al., 2017). The whorled interpretation is dominating in recent literature, but, for example, Padgett (2007) describes spirally arranged appendages enclosing a compound ovary in *Nuphar*. Even within the whorled interpretation, details of organ arrangement such as the number of petal whorls remain questionable. In the present study, we are making at attempt of resolving these problems. Because flower development is strongly related to flower positioning of the rhizome, both flower and rhizome development are covered here.

## Materials and Methods

Material of *Nuphar lutea* L. (growing tips of rhizomes or entire rhizomes) was collected in river Usmanka, near the Biological Teaching and Scientific Centre ‘Venevitinovo’ of Voronezh State University (Novousmansky distr., Voronezh prov., Russia) in June 2009 (voucher: *Sokoloff s.n.*, MW1063500) and in Moskva River near village Lutsino (Odintsovsky distr., Moscow prov., Russia) in June-September 2012 (voucher: *Sadovnikova s.n.*, MW1063501). The material of *N. pumila* (Timm) DC. was collected in Vashutinskoe lake (Pereslavl distr., Yaroslavl prov., Russia) in July 2013 (voucher: *Sadovnikova s.n.*, MW1063499).

All material was fixed in 70% ethanol. For scanning electron microscopy (SEM), specimens were dissected under a stereomicroscope and then dehydrated in alcohol-acetone series, critical-point dried in liquid CO_2_ using a Hitachi HCP-2 critical point dryer, mounted on aluminum stubs, coated with gold or platinum using an Eiko IB-3 ion-coater and observed using a JSM-6380LA SEM and CamScan 4 DV at the Department of electron microscopy at the Faculty of Biology, Moscow State University. Tips of 25 rhizomes of *N. lutea* (all from Moskva River) and of 10 rhizomes of *N. pumila* were used for SEM investigations.

Some flowers have been sectioned anatomically after documenting their morphology using SEM. These dry samples were transferred into 70% ethanol through 100% acetone and then processed using standard anatomical methods (Barykina et al., 2004) with paraplast embedding and serial sectioning at a thickness of 15 μm using the HM 355S Automatic Microtome (Thermo Fisher Scientific). A microtome knife sharpener KS-250 (Thermo Fisher Scientific) was used. The sections were stained in picroindigocarmine and carbolic fuchsine using a Varistain GEMINI ES Automated Slide Stainer and mounted in Bio Mount (Bio-Optica, Milano). Sections were examined and images were taken using a Zeiss Axioplan microscope.

Entire rhizomes were analyzed with respect to the arrangement of all lateral organs and scars of all abscised organs in older parts of the rhizomes. Nineteen entire rhizomes of *N. lutea* were used for quantitative study of organ arrangement. The data set is provided in Supplementary Data 1. Methods used for visualization of the quantitative data are explained in the caption of Figure 3. To study possible seasonality in organ initiation, four plants of *N. lutea* were selected. Rhizome of each plant has been marked in early June of 2012 by a metallic ring applied just below the first leaf appeared in that vegetation season. These rhizomes were collected at the end of the vegetation season in October 2012 and analyzed with respect to organ arrangement.

## Results

### Rhizome Morphology and Flower Arrangement

The description of rhizomes is based on *N. lutea*. We had less material on *N. pumila*, but the features described below were found in this species, too, except the occurrence of the collateral groups of the rhizome branches. Also, we did not study young rhizomes before the first branching in *N. pumila*.

**FIGURE 1:**
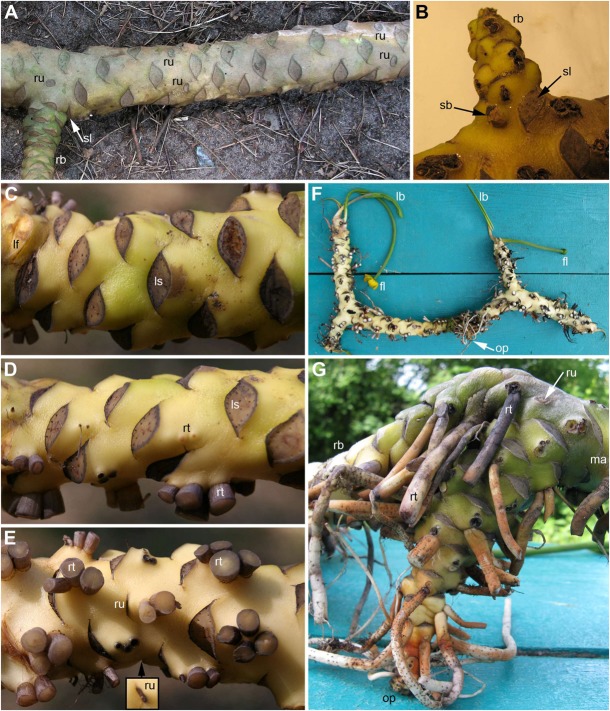
Rhizomes of *Nuphar lutea*. **(A)** Dorsal view of rhizome with a lateral branch. Scars of five reproductive units (RUs) are visible. Four of them form pairs and the fifth is associated with the lateral branch. **(B)** Detail of rhizome with a lateral branch and a supernumerary bud in axil of the same subtending leaf. Dorsal **(C)**, lateral **(D)**, and ventral **(E)** views of a rhizome. The roots were cut off before taking the images. Inset shows another view of RU scar that is only slightly visible in panel **(E)**. **(F)** Ventral view of entire branching rhizome with the oldest part remaining (roots and fully expanded leaves cut off). **(G)** Close up of the oldest, vertical part of the rhizome illustrated in panel **(F)**. This part was formed when the plant was young. The first branching event was associated with a shift to dorsiventrality in both the branch and the main axis. fl, flower; lb, leaf blade; lf, cut leaf base (this leaf of the current season was still attached to the plant); ma, main axis; ls, leaf scar; op, oldest part of the rhizome; sl, scar of subtending leaf of rhizome branch; rb, rhizome branch; rt, root; ru = scar of RU; sb, supernumerary lateral bud.

**FIGURE 2:**
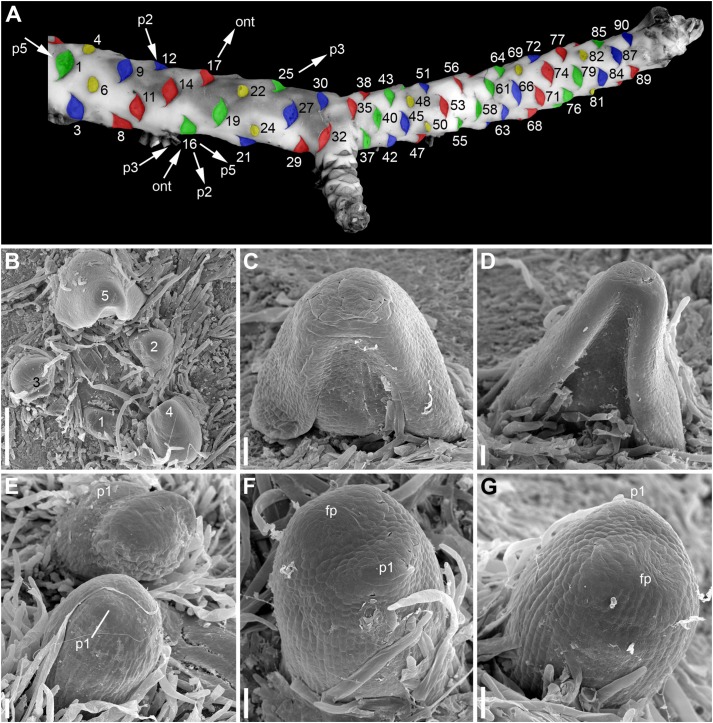
Rhizome morphology and early development of its lateral organs in *Nuphar lutea* (**A**, photo, **B–G**, SEM). **(A)** Dorsal view of rhizome with artificially colored scars of abscised lateral organs. Yellow, scars of RUs, green, blue and red, scars of vegetative leaves. The three colors are used to show that the leaves can be viewed as forming three parastichies, with RUs taking part in formation of these parastichies. The lateral organs of the rhizome are numbered in an acropetal order starting arbitrary from the first visible leaf scar (actually, the rhizome is longer than shown here). The leaf 32 is a subtending leaf of the rhizome branch. There is a RU in the position 34, which is not visible here because it is on the ventral side of the rhizome. Using the leaf 16 as an example, the arrows indicate the four kinds of spirals that can be drawn through each lateral organ of the rhizome. ont = ontogenetic spiral; p2, a spiral that belongs to a set of two parastichies (it comprises all organs with even numbers, the other spiral of this set includes all organs with odd numbers); p3, a spiral that belongs to a set of three parastichies (it includes all leaves colored green plus RUs 4, 22, 34, 82); p5, a spiral that belongs to a set of five parastichies (it includes organs 1, 6, 11, 16, 21, 26, 31, 36, 41, 46, 51, 56, 61, 66, 71, 76, etc.). The spiral p3 has the same direction as the ontogenetic spiral while the spirals p2 and p5 have another direction. B, top view of shoot apex with leaves at different developmental stages (numbered starting from the youngest leaf). **(C,D)** Ventral views of leaf primordia at successive stages (the leaf in **(D)** is the leaf 5 in **B**). **(E)**, RUs at distances of 4 (below) 6 (above) plastochrons from the rhizome apex. **(F,G)** Two views of the same RU at distance of 4 plastochrons from the rhizome apex. **(F)** Oblique abaxial view. **(G)** Adaxial view. fp, flower primordium; p1, the first scale-like phyllome. Scale bars = 300 μm in **(B)**, 50 μm in **(C–G)**.

The one-flowered reproductive units (RUs) with long-pedicellate flower as well as the long-petiolate foliage leaves with floating blades are spirally arranged along a massive thick creeping monopodial rhizome. The RUs are not located in the axils of the foliage leaves (Figures 1A, 2A). There are no cataphylls directly attached to the rhizome. The RUs appear to ‘replace’ the foliage leaves in some positions of the ontogenetic spiral of phyllotaxis, or, in other words, the RUs are included in the same spiral as the foliage leaves (Figure 2A).

The rhizomes are dorsiventrally flattened, except in young plants. Since the rhizome apex is apparently oblique (displaced toward the dorsal side of the rhizome), the leaves are obliquely inserted on the lateral sides of the rhizome (Figure 1D) and transversally inserted on the dorsal (Figure 1C) and ventral (Figure 1E) sides. As a result, the ontogenetic spiral and the parastichies are somewhat ‘deformed’ relative to their ideal shapes: they are ‘shifted forward’ on the ventral side and ‘shifted backward’ on the dorsal side. According to Raciborski (1894a), rhizomes that grow very deep in the ground (e.g., when flooded with earth due to the slippage of a brook bank), are growing straight up and are almost completely built radially.

The arrangement of the leaves and the RUs along the rhizome follows the Fibonacci pattern. Assuming that organs 6 and 90 in Figure 2A both occupy positions close to dorsal median, we calculated an empirical divergence angle as 137.2°, which is very close to the theoretical value for the Fibonacci pattern (137.5°). Direction of the ontogenetic spiral is either clockwise or anticlockwise. Sets of 2, 3 and 5 parastichies can be recognized (Figure 2A). As predicted by the Fibonacci pattern, the parastichies forming the sets of 2 and 5 spirals have a direction that is opposite to that of the ontogenetic spiral, while those forming the set of 3 spirals follow the direction of the ontogenetic spiral (Figure 2A).

The rhizomes remain undamaged during several years after abscission of foliage leaves and flowers. The positions of all abscised organs can be easily inferred from their scars (Figures 1, 2A) that remain clear throughout the life of the rhizome. The pedicel scars are circular or elliptic (Figures 1A,E,G, 2A). The leaf scars are elliptic with acute left and right angles (Figures 1A,C–E, 2A). Adventitious roots arise, usually in groups of 2-4, below leaf bases on the ventral side of the rhizome (Figures 1D,E). They are initiated in the same positions but arrested at early stages on the lateral sides of the rhizome (Figure 1D). The roots are absent on the dorsal side (Figure 1C).

**FIGURE 3:**
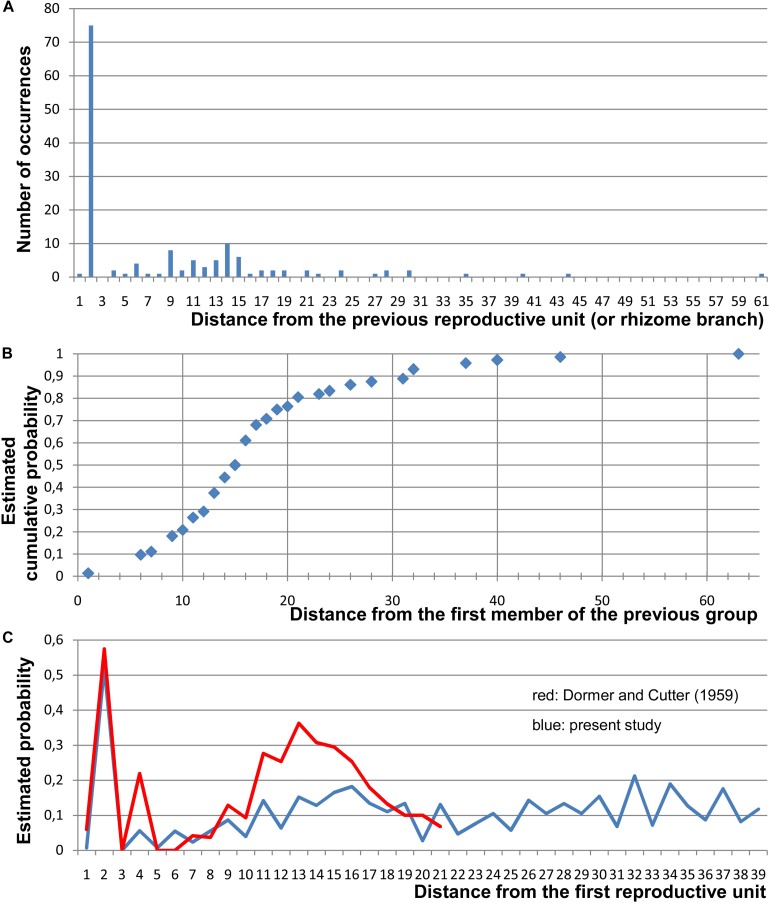
Quantitative data on the arrangement of RUs and rhizome branches in *Nuphar lutea*. For this study, 19 entire rhizomes from the locality in Voronezh province were analyzed and positions of all organs along the ontogenetic spiral were documented. **(A)** Occurrence of different distances (measured in the number of organs along the ontogenetic spiral) between positions of successive lateral RUs and/or positions with rhizome branches (collectively called ‘branching sites’). In each rhizome, a distance from each branching site to the next branching site has been measured. Total numbers of occurrences of various distances across all 19 rhizomes are plotted here. In more than 1/2 of the instances, the distance was 2 (i.e., the adjacent branching sites occurred in the positions N and N + 2). **(B)** Occurrence of different distances between the groups, each group containing one, two (N, N + 2), or three (N, N + 2, N + 4) branching sites. Distances from the first site of a group to the first site of the nearest subsequent group have been measured. Estimated cumulative probability rather than absolute numbers of occurrences is shown here. **(C)** Comparison between the present study and the study of Dormer and Cutter (1959). Given that a branching site is present at position 0, each graph shows the frequence of the occurrence of a branching site at each subsequent position of a rhizome. For this analysis, position of the first branching site of a rhizome was treated as 0 and distances to all subsequent branching sites (BS) and non-branching sites (NB) of the rhizome were recorded. Then the second branching site of the rhizome was assigned as position 0 and the same calculation was performed. Following Dormer and Cutter (1959), estimated probability was calculated as BS/(BS + NB) for each distance across all records taken from all rhizomes. Note that Dormer and Cutter (1959) removed positions with lateral rhizomes from their data set (treated these positions as uncertain), but lateral rhizomes were rare in their material.

Distribution of the RUs and the lateral shoots (= rhizome branches) along the length of the rhizome follows certain regularities (Figure 3). The RUs tend to form pairs (Figures 1A, 2A). The two RUs of a pair are separated by a foliage leaf in the ontogenetic spiral of phyllotaxis, so that the positions N and N + 2 are occupied by the RUs (Figure 2A) and the position N + 1 (as well as N-1 and N + 3) has a foliage leaf. The organs are numbered in the sequence of their initiation. For example, in the rhizome in Figure 2A, three such pairs of reproductive units can be seen (in positions 4 and 6, 22 and 24, 48 and 50). The two RUs of a pair are spatially close to each other (Figures 1A, 2A). They hold adjacent positions in a parastichy (namely, in one of the parastichies forming a set of two). Much less frequently, the RUs appear singly (not accompanied by another RU in the position N + 2) or in triplets in positions N, N + 2, N + 4. For example, in the rhizome in Figure 2A, there is a RU in the position 69, but organs 67 (on the ventral side, not shown) and 71 are leaves. Occurrence of two RUs in adjacent positions of the ontogenetic spiral (N, N + 1) is extremely rare (Figure 3A), but one of these rare instances can be seen in Figure 2A (in positions 81 and 82). The number of positions of the ontogenetic spiral between the pairs of the RUs (or single RUs or their triplets) is highly variable (Figure 3B), and there is no obvious correlation with any other parameter. In particular, the RUs can be located on any side of a rhizome (dorsal, lateral or ventral, Figures 1A,E). Indeed, out of the positions of 138 RUs counted in our quantitative study, 69 were associated with roots and 69 had no roots near their bases. Observations on annual dynamics of the rhizome development demonstrated the absence of any clear correlation between year seasons and initiation of the RUs. A rhizome produces 25–35 foliage leaves and typically more than one pair of RUs a season (Table 1). Our direct observations showed no obvious seasonality in the activity of the rhizome apex (Table 1). In the absence of direct observations it is almost impossible to detect the boundaries between successive years in long perennial rhizomes of *Nuphar*. In our quantitative study based on 19 entire rhizomes, 1/2 of all measured distances between RU groups (or branch + unit groups or single units) was in the interval between 11 and 19 with the median value 15 (Figure 3B). Based of our field experiments, these figures have nothing to do with potential seasonality of rhizome growth.

**TABLE 1:** Seasonal dynamics of rhizome development in *Nuphar lutea*. Four plants were selected for the experiment.

Rhizome 1. 1 2 3 4 5 6 7 8 (9) 10 11 12 13 14 15 16 17 18 19 20 21 22 23 24
Rhizome 2. 1 2 3 4 5 6 7 8 9 10 11 12 13 (14) 15 (16) 17 18 19 20 21 22 23 24 25 26
Rhizome 3. 1 2 3 (4) 5 (6) 7 8 9 10 11 12 13(rhizome branch in axil of this leaf) 14 (15) 16 17 18 19 20 21 22 23 24 25 26 27
Rhizome 4. 1 2 3 4 (5) 6 (7) 8 9 10 11 12 13 14 15 16 17 18 19 20 21 22 23 24 25 26 27 28 29 (30) 31 (32) 33


*Rhizome of each plant has been marked in early June of 2012 by a metallic ring applied just below the first leaf appeared in that vegetation season. These rhizomes were collected at the end of the vegetation season in October 2012 and analyzed with respect to organ arrangement. Diagrams of these rhizomes are provided below. Numbers are organ numbers in the ontogenetic spiral of the rhizome. Numbers without parentheses are vegetative leaves. Number in parentheses are RUs (flowers). Underlined numbers indicate the occurrence of adventitious roots associated with the organ base. Occurrence of a rhizome branch is indicated in parentheses after the number of its subtending leaf. Only organs fully expanded in the current season are considered.*

**FIGURE 4:**
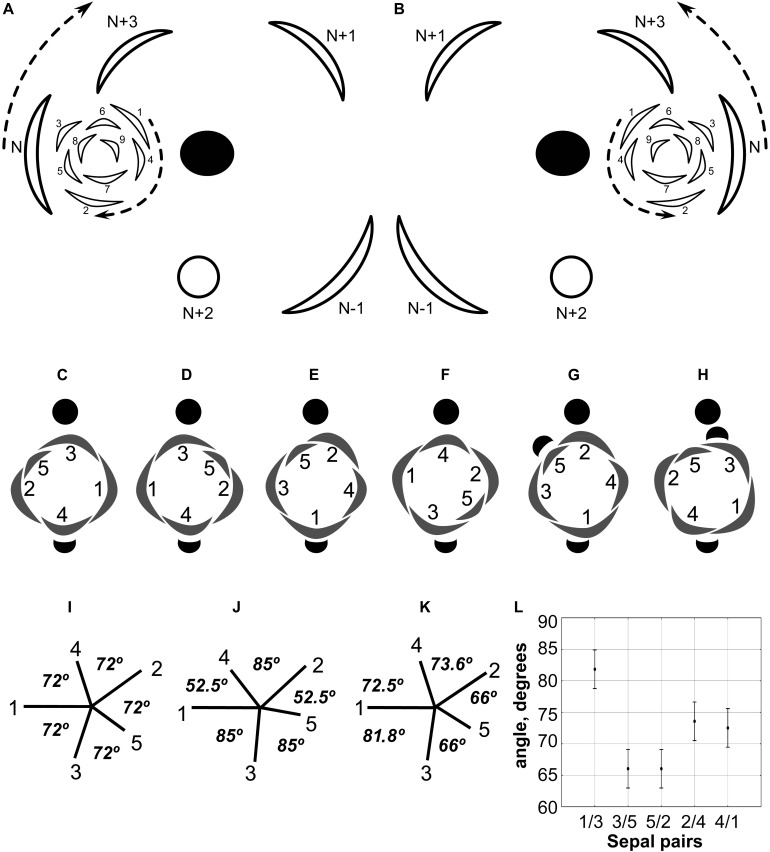
**(A,B)** Diagrams showing the patterns of rhizome branching and the most common position of the associated RU in *Nuphar lutea*. **(A)** Ontogenetic spirals clockwise. **(B)** Ontogenetic spirals anticlockwise. Black ellipse, rhizome axis that is slightly dorsiventrally flattened; arcs with thick lines, leaves of the main axis; arcs with thin likes, leaves of the branch (1–9); open circle, RU with flower; arrows, direction of ontogenetic spirals; N-1, N, N + 1, N + 2, N + 3, positions in the ontogenetic spiral of the main axis, N is the subtending leaf of the branch and N + 2 is a RU. **(C–H)** Diagrams of all observed patterns of sepal and scale-like phyllome arrangement in *N. lutea*. The most common type in two mirror forms, with clockwise **(C)** and anticlockwise **(D)** sequence of sepal arrangement (and initiation). **(E–H)** Rare types. **(I–K)** Patterns sepal arrangement in calyx with five sequentially initiated sepals. 1,2,3,4,5, sepal numbers. Angles between adjacent sepals are indicated. **(I)** All sepals forming a whorl (theoretical prediction). **(J)** Sepals forming a Fibonacci spiral (theoretical prediction). **(K)** Pattern observed in *Nuphar lutea*, mean values of observed angles are indicated. **(L)** Mean values and confidence intervals for angles between adjacent sepals based on our measurements in 23 flowers of *N. lutea* (see Supplementary Data 2, for the data set).

Rhizome branching is always axillary. The subtending leaf of lateral rhizome does not differ from other foliage leaves. Formation of lateral rhizomes is in most cases associated with flower formation in the following way (Figures 4A,B): instead of a pair of RUs in positions N, N + 2, a lateral shoot is formed in the position N and a RU is formed in the position N + 2. Out of 24 instances of rhizome branching found in our quantitative study, 22 had a lateral shoot in the position N and a RU in the position N + 2, as outlined above (Figures 4A,B). In two instances, a lateral shoot in the axil of leaf N was accompanied by a RU in the position N-2 (i.e., the lateral shoot was found where the *second* unit of a pair could be expected).

Shoot branching almost always occurs at the lateral sides of the rhizome, though the RUs are present also on the dorsal and ventral sides. Out of many rhizomes examined, only one instance of branching on the dorsal rhizome side was observed. Unfortunately, this particular part of the rhizome was an old one, with the evidence of decay of some organs, so that it was impossible to draw a complete picture of arrangement of all organs. In two instances (in different individual plants), two rhizome branches were observed in the axil of the same subtending leaf on a lateral side of the rhizome. In both cases, the two branches were located side by side to each other, indicating their development from a collateral group of buds (Figure 1B). The larger of the two branches of a pair was in a cathodic position relative to the subtending leaf (the side that is closer to the beginning of the ontogenetic spiral) while the smaller of the two branches was in an anodic position (closer to the end of the ontogenetic spiral). Like in single branches, in both observed instances, a collateral group in the position N was associated with a RU located in the position N + 2 (in the rhizome in Figure 1B, the associated RU is on the ventral side).

The phyllotaxis of the lateral branches starts with foliage leaves (there are no cataphylls). The first leaf is in an anodic position relative to the subtending leaf, the second leaf is in a cathodic position, the third leaf is in an adaxial position being slightly shifted toward the anodic side, then the phyllotaxis continues following the Fibonacci pattern (Figures 4A,B). As a result (Figures 4A,B), the direction of the ontogenetic spiral of the lateral rhizomes is always the same as in the maternal rhizome (also in both branches of the collateral groups). The shoot chirality (clockwise or anticlockwise) is established at seed germination and conserved throughout the life of the entire plant.

The first rhizome branching in plant ontogeny occurs along with formation of the first flower (Figures 1F,G). The oldest part of the rhizome (before the first branching) is upright and not dorsiventrally flattened, with adventitious roots present below all leaf bases (Figure 1G). After the first branching, dorsiventrality is conspicuous in the main well as in the lateral rhizome (Figure 1G).

Rhizome branching is sylleptic, i.e., the branch growth takes place simultaneously with continuation of growth of the main axis. Dormant buds are absent. The buds are totally absent in the axils of all foliage leaves except those subtending sylleptic rhizome branches as described above. The absence of buds is documented by examination of the external morphology, anatomy and development, including observations of shoot apices using SEM and extensive search of young branches on old rhizomes.

### Reproductive Units and Flowers

**FIGURE 5:**
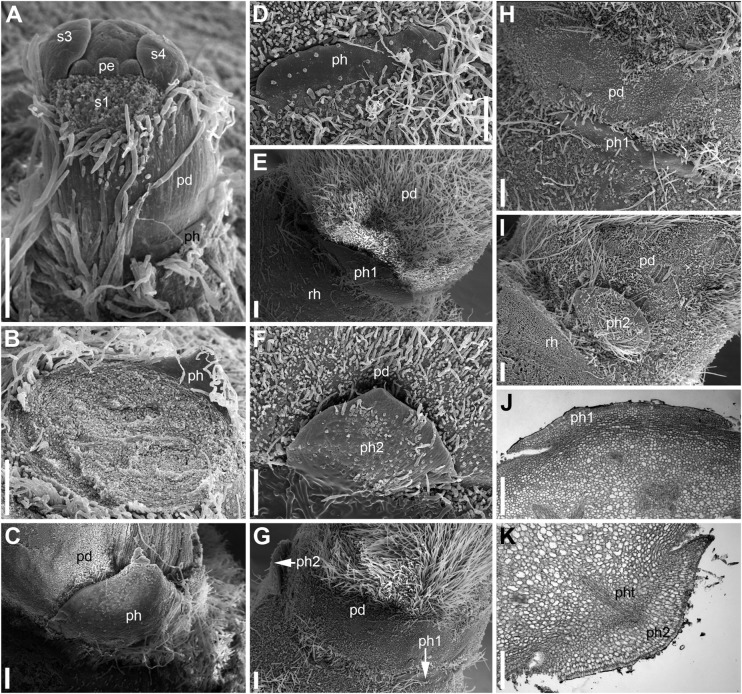
Scale-like phyllomes at the pedicel base of *Nuphar lutea* (**A–I**, SEM; **J,K**, LM). The densely spaced hairs are partially removed in panels **(C–K)**. **(A)** Pre-anthetic RU with single scale-like phyllome. The floral pedicel is yet short. **(B–K)** Anthetic RUs. **(B–D)** RUs with single scale-like phyllome showing variation of its shape. The floral pedicel is removed in **(B)**, so that the phyllome is seen from its adaxial side. **(C,D)** Abaxial view of the phyllome. **(E,F)** RU with two phyllomes (see diagram in Figure 4H). **(E)** Phyllome 1. **(F)** phyllome 2. **(G–K)** Another RU with two phyllomes (see diagram in Figure 4G). **(G)** Side view showing both phyllomes. **(H,J)** Phyllome 1. **(I,K)** Phyllome 2. pd, pedicel; pe, petal; ph (ph1, ph2), scale-like phyllomes; pht, phyllome trace; rh, rhizome; s1, s3, s4, sepals in sequence of their initiation. Scale bars = 300 μm in **(A–K)**.

Each RU consists of a short (about 1 mm long) cylindrical common base, a very long cylindrical pedicel bearing a flower situated at the water surface and one or two scale-like phyllomes (Figure 5) at the junction of the cylindrical common base and the pedicel. Almost all examined flowers of *N. lutea* possessed only one phyllome, and its position relative to the rhizome was abaxial (Figures 4C–F). The phyllome shape is triangular with acute tip (Figures 5B,C) to short and wide with obtuse tip (Figure 5D). We found only two flowers with a pair of phyllomes at the base of the pedicel in *N. lutea* (Figures 5E–K). In these two flowers, one phyllome was in the abaxial position, while another one was nearly adaxial, but its position slightly differed between the two RUs where it was observed (Figures 4G,H). In both units with two phyllomes, the phyllome 2 had a narrower base than phyllome 1 (Figures 5E,F,H,I) and in one of the two instances, only the phyllome 2 was vascularized (Figure 5K). As the phyllomes are short, they are usually hidden by the long hairs that cover all surrounding organs.

**FIGURE 6:**
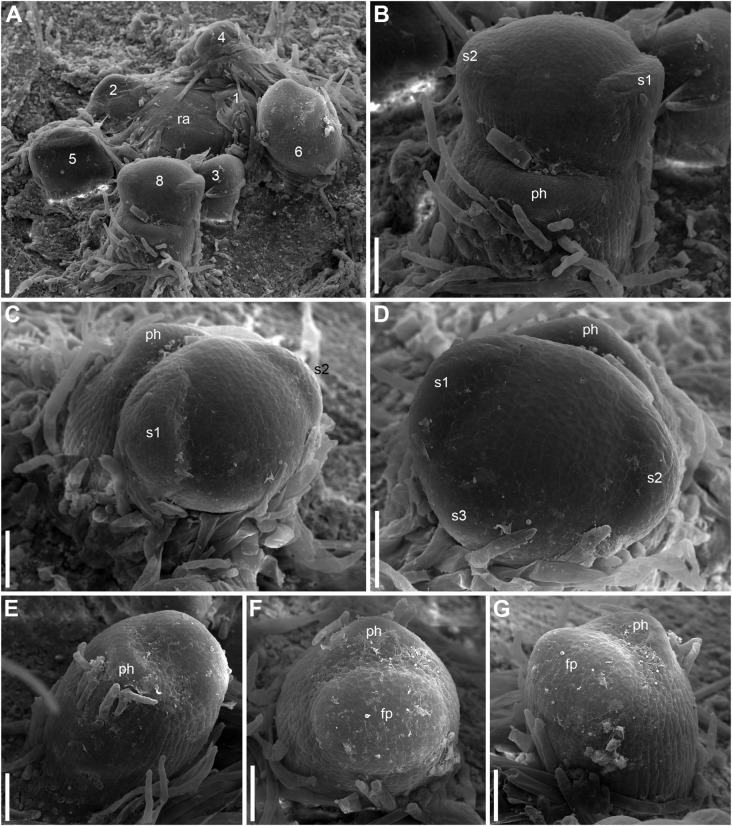
Early development of RUs in *Nuphar lutea* (SEM). **(A)** Rhizome apex surrounded by organs of different age numbered starting from the youngest one. These numbers can be viewed as organ age measured in plastochrons. Organ 7 (leaf) was removed during dissection. 6 and 8 are RUs. **(B–D)** different views of the RU 8 from **(A)**. **(E–G)** Different views of the RU 6 from **(A)**. fp, floral primordium; ph, scale-like phyllome; ra, rhizome apex, s1, s2, s3; sepals in sequence of their initiation. Scale bars = 100 μm in A–G.

**FIGURE 7:**
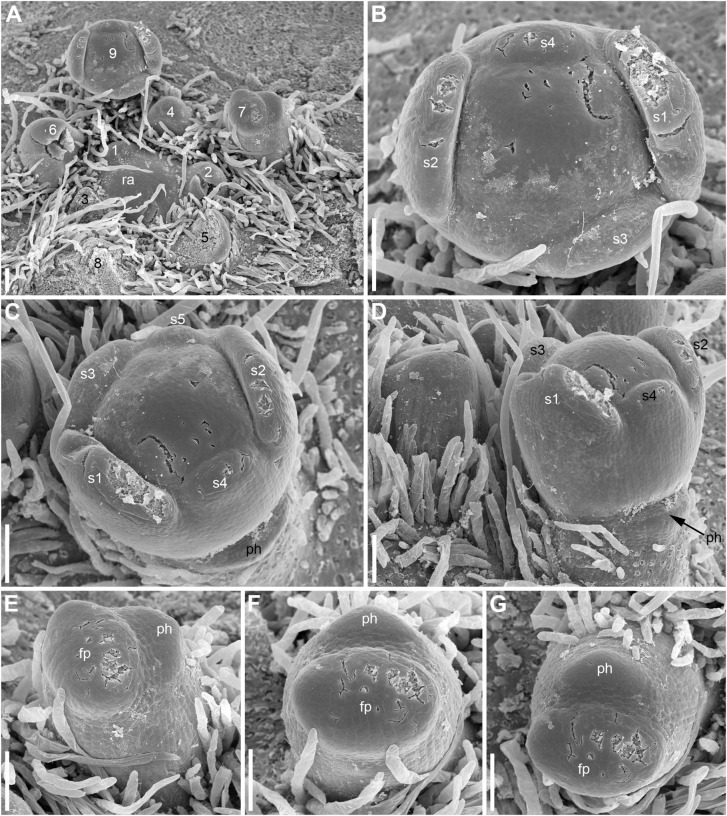
Early development of RUs in *Nuphar lutea* (SEM). **(A)** Rhizome apex surrounded by organs of different age numbered starting from the youngest one. 7 and 9 are RUs. **(B–D)** Different views of the RU 9 from **(A)**. **(E–G)** Different views of the RU 7 from **(A)**. fp, floral primordium; ph, scale-like phyllome; ra, rhizome apex, s1–s5; sepals in sequence of their initiation. Scale bars = 100 μm in **(A–G)**.

**FIGURE 8:**
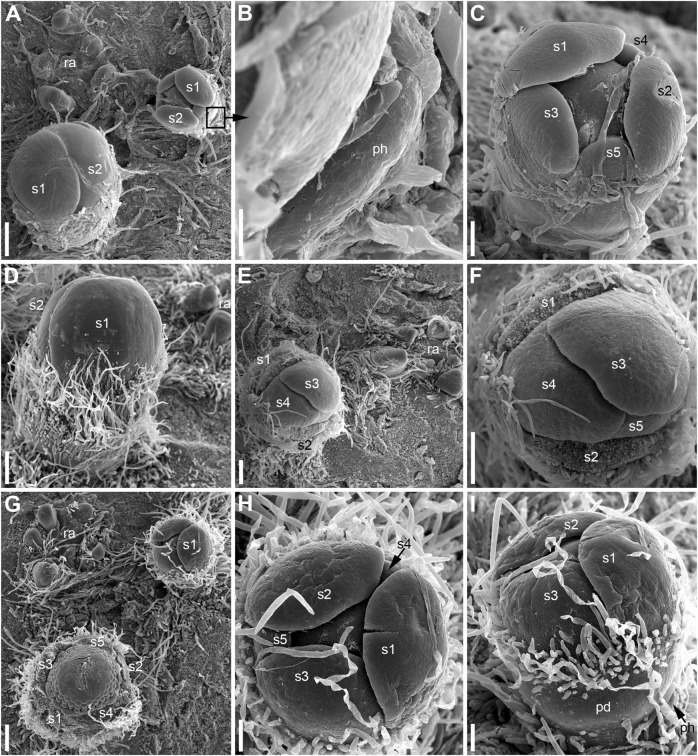
Patterns of flower orientation and calyx aestivation and in *Nuphar lutea* (SEM). **(A–F)** Flowers with the most common type of orientation (see Figures 4C,D). **(A)** Two flowers in positions N, N + 2 on the rhizome. The older flower has an anticlockwise sepal arrangement (as in Figure 4D), the younger flower has a clockwise sepal arrangement (as in Figure 4C). **(B)** Much enlarged detail of **(A)** (marked by a frame in **A**) showing the occurrence of a scale-like phyllome associated with the younger flower. **(C)** The younger flower from **(A)**. **(D)** Flower at about the same stage as the older flower in **(A)**, but with clockwise sepal arrangement. **(E)** Flower with sepals 1 and 2 removed. **(F)** Close up of **(E)**, sepal arrangement anticlockwise (as in Figure 4D). **(G)** Two flowers with anticlockwise sepal arrangement and their position relative to rhizome apex. The younger flower with sepal 1 abaxial (as in Figure 4E). **(H,I)** Two views of the younger flower from **(G)**. pd, pedicel; ph, scale-like phyllome; ra, rhizome apex; s1–s5 sepals numbered in the sequence of their initiation. Scale bars = 300 μm in **(A,D–G)**, 30 μm in **(B)**, 100 μm in **(C,H,I)**.

Developmental data are only available for RUs of *N. lutea* with single scale-like phyllome (Figures 6, 7, 8A,B). The earliest evidence of scale-like phyllome can be seen in RUs at distance of 6 plastochrons from the rhizome apex (Figures 6A,E–G). At this stage, the cylindrical common base of RU is already longer than the crescent-shaped phyllome primordium. The phyllome primordium is abaxial relative to the rhizome apex. On the adaxial side of RU, a flower primordium can be seen (Figures 6F,G). The flower primordium does not look like a direct continuation of the common base of RU because of its displacement on the adaxial side, its elliptic (elongated transversally) outline and clearly demarcated borders (Figure 6F). Similar picture can be seen at slightly older stage (7 plastochrons from the rhizome apex, Figures 7A,E–G). The flower primordium is even more transversally elongated (Figure 7F) and its borders are clearly demarcated (Figure 7E). In these early stages, the width of the scale-like phyllome is compatible to the width of the floral primordium and it is only slightly shorter than the latter (Figures 6E–G, 7E–G). With subsequent flower development, the common stalk of the RU and the scale-like phyllome exhibits only limited growth and become hidden by surrounding structures. Already at the stage with all sepals initiated, special efforts are needed to document the occurrence of the phyllome (Figures 8A,B).

Hairs first develop on the abaxial side of the common stalk of RU (Figures 6E, 7G) and then on its adaxial side (Figure 6C). Hairs on the pedicel first appear in its distal part (Figures 8C,I), probably because the intercalary growth is localized in its proximal part.

**FIGURE 9:**
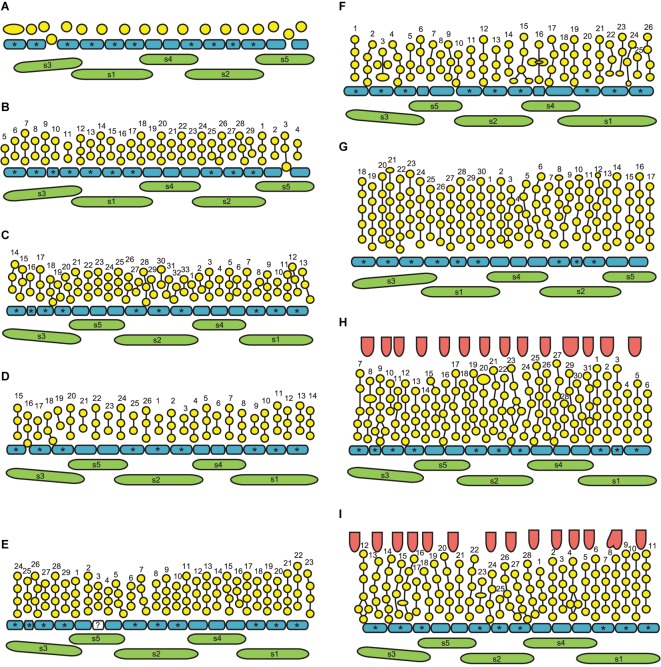
Flower diagrams of *Nuphar lutea*. Here and in Figure 10, the diagrams are prepared in a special way. Because the organs are so numerous and the receptacle is so convex, we found it more convenient to present schematical side views (rather than top views) of the flowers. Each flower is ‘cut’ through a radius near sepal 3 and then ‘unrolled’ to place all organ positions in the same plane. **(A–F)** Flowers at successive stages of androecium development. **(G)** Flower with androecium initiation completed, but gynoecium not yet formed. **(H,I)** Flowers with all organs initiated. green, sepals (labeled s1–s5 following their aestivation/initiation pattern); blue, petals (asterisks indicate the petals occurring in the sectors of the outer whorl sepals, s1–s3); yellow, stamens; vertical lines, an attempt of recognized stamen orthostichies; red, carpels. The putative orthostichies are numbered starting from an arbitrary point. Question mark indicates an organ that cannot be precisely identified as stamen or petal at this developmental stage.

**FIGURE 10:**
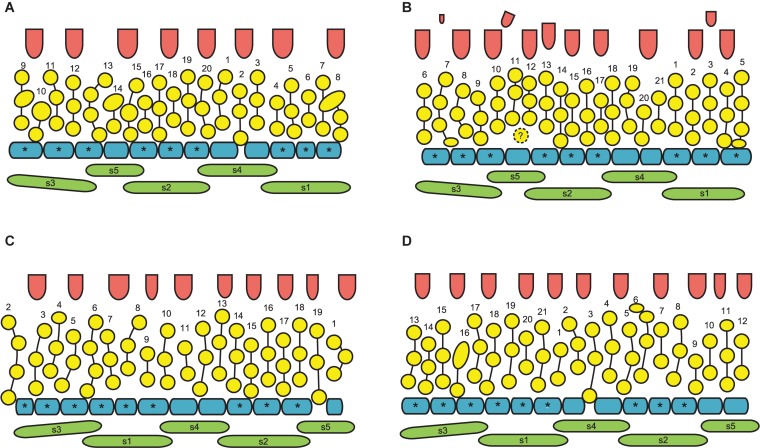
**(A–D)** Flower diagrams of *Nuphar pumila*. See Figure 9 for explanations. The second whorl of the gynoecium is only illustrated when more than one carpel was found **(B)**.

The flowers are cup-shaped, with five free rounded sepals, numerous (13–16 in *N. lutea* and 12–13 in *N. pumila*) free narrow nectariferous petals, very numerous (97–150 in *N. lutea* and 55–59 in *N. pumila*) cuneate stamens and a syncarpous superior gynoecium of 14–17 carpels in *N. lutea* and of 8–11 carpels in *N. pumila* (Figures 9, 10). The sepals are convex and much longer and wider than the petals and stamens, forming a protection over the other organs in the bud. Petals and stamens are inserted at the convex receptacle around the club-shaped ovary.

### Calyx

**FIGURE 11:**
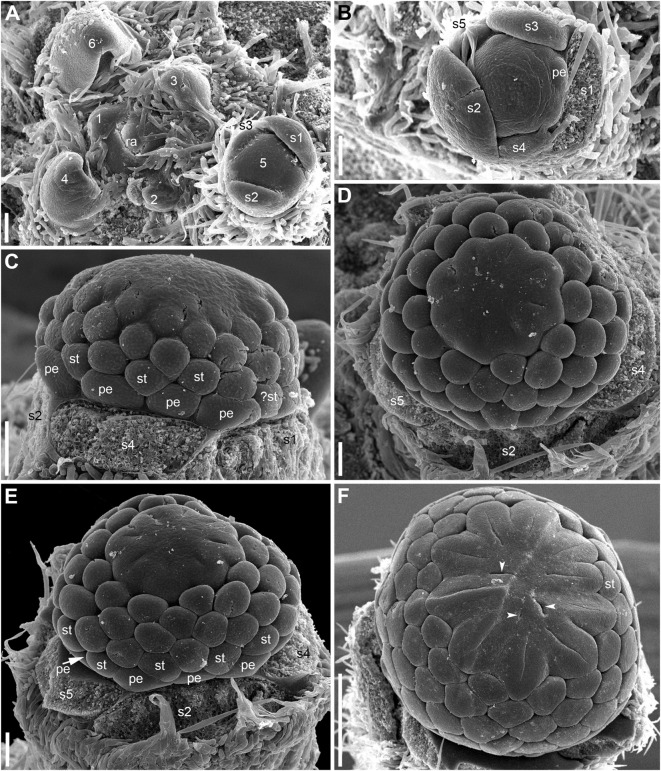
Flower development in *Nuphar pumila* (SEM). **(A)** Flower before petal initiation and its position relative to rhizome axis. Organ age measured in plastochrons is indicated by arabic figures. **(B)** Flower before stamen initiation. **(C)** Flower before carpel initiation. Labels indicate the apparent outer whorl stamens. **(D,E)** Two views of flower with gynoecium just initiated. All carpels are in a single whorl. Diagram of this flower is in Figure 10A. Labels in E indicate the apparent outer whorl stamens. **(F)** Flower that is older than in panels **(D,E)**. Its diagram is in Figure 10B. Carpels are in two whorls, the second whorl is incomplete and consists of three carpels (arrowheads). The only labeled stamen is one of the innermost stamens and a carpel is located right on its radius. Therefore, this carpel is shifted toward the center of the flower and its position is intermediate between the outer and the inner whorl. It is possible that the asymmetry of the gynoecium caused by this shift triggered the appearance of the inner whorl carpels. It is likely that transference of positional information from androecium to gynoecium was essential in development of this flower, but in our view there is no way of testing a hypothesis that any mechanical pressure (Ronse De Craene, 2018) took place here. pe, petals; ra, rhizome apex; s1–s5, sepals in the sequence of their initiation; st, stamens. Scale = 100 μm in **(A–E)**, 500 μm in **(F)**.

The calyx always has a quincuncial aestivation (Figures 4C–H, 8A,C,F,H). Clockwise (Figures 4C,F,H, 8C,D, 11A,B) and anticlockwise (Figures 4D,E,G, 8F,H) types of the quincuncial aestivation can be recognized. Both types can be found in different flowers of the same plant, sometimes even in the two RUs in the positions N and N + 2 along a rhizome (Figure 8A).

In RUs with single scale-like phyllome, the flowers usually have the two outermost sepals in transversal-abaxial positions, next two larger sepals in almost median positions and an innermost and the smallest sepal in lateral-adaxial position (Figures 4C,D, 6B,D, 7A–D, 8A,D,E, 11A). In this common type of flower orientation, the sepal 3 (the one with one margin external to its adjacent sepal and the other margin internal to another adjacent sepal) is close to an adaxial position (Figures 4C,D, 7D, 8E). A similar pattern of sepal arrangement was also found in one of the two examined RUs bearing two scale-like phyllomes (Figure 4G). The following exceptions from the typical pattern of flower orientation have been documented: (1) we found three flowers with the outermost sepal abaxial and the sepal 3 transversal-adaxial (two of them with single scale-like phyllome – Figures 4E, 8G–I – and one with two phyllomes – Figure 4G), and (2) a flower with the outermost sepal transversal-adaxial and the sepal 3 transversal-abaxial in a RU with single scale-like phyllome (Figure 4F). In the latter case, flower orientation was inverted with respect to the typical condition (Figures 4C,D).

Sepal initiation pattern corresponds to the pattern of sepal aestivation, but the process of sepal initiation is very rapid. We only have clear evidence that sepals 1-3 appear before the sepals 4 and 5. In *N. lutea* (for which we had more material), no sepal primordia was observed in flowers until the distance of 7 plastochrons from the rhizome apex unless the transversal elongation of the floral meristem can be interpreted as the earliest manifestation of sepals 1 and 2 (Figures 7E,F). At the distance of 8 plastochrons from the rhizome apex, sepals 1 and 2 are well initiated and a smaller primordium of sepal 3 can be seen (Figures 6A–D). At the distance of 9 plastochrons from the rhizome apex, all five sepals are initiated in *N. lutea* (Figures 7A–D). In *N. pumila*, similar stage of calyx development was found at the distance of only 5 plastochrons from the rhizome apex (Figure 11A).

The first three sepal primordia are pronouncedly crescent-shaped whereas the last two are almost rounded in outline and only slightly extended along the apex circumference. After initiation, the sepals grow rapidly to enclose the inner parts of the developing flower. At later stages of flower development, sepals 1 and 2 cover three other petals completely (Figures 8A,D).

Angles between sepals were measured in 23 flowers (Figures 4K,L). Mean angle between the sepals 1 and 2 was greater than that between the sepals 2 and 3 (about 146° and 132°, respectively). The sepal 4 appeared almost in the middle between the sepals 1 and 4 (mean angles 72.5° and 73.6°, and the difference between these values is not significant, Figure 4L). The sepal 5 appeared just in the middle between the sepals 2 and 3 (Figures 4K,L).

### Corolla

**FIGURE 12:**
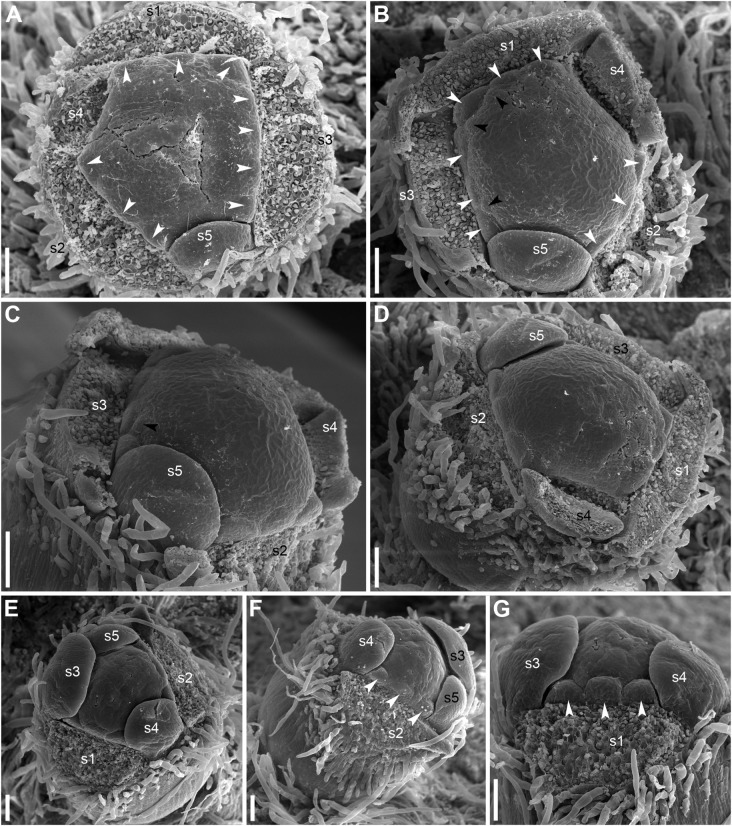
Early corolla development in *Nuphar lutea* (SEM). **(A)** Top view of flower with four of five sepals removed. Note the absence of petals in sectors of the sepals 4 and 5. **(B–D)** Three different views of another flower with four sepals removed. Petals are yet absent in the sectors of the sepals 4 and 5. Note the appearance of the first stamens in the sectors of the sepals 1 and 3. **(E–G)** Three different views of flower with two sepals removed. s1–s5, sepals in sequence of their initiation; white arrowheads, petals; black arrowheads, stamens. Scale bars = 100 μm in **(A–G)**.

**FIGURE 13:**
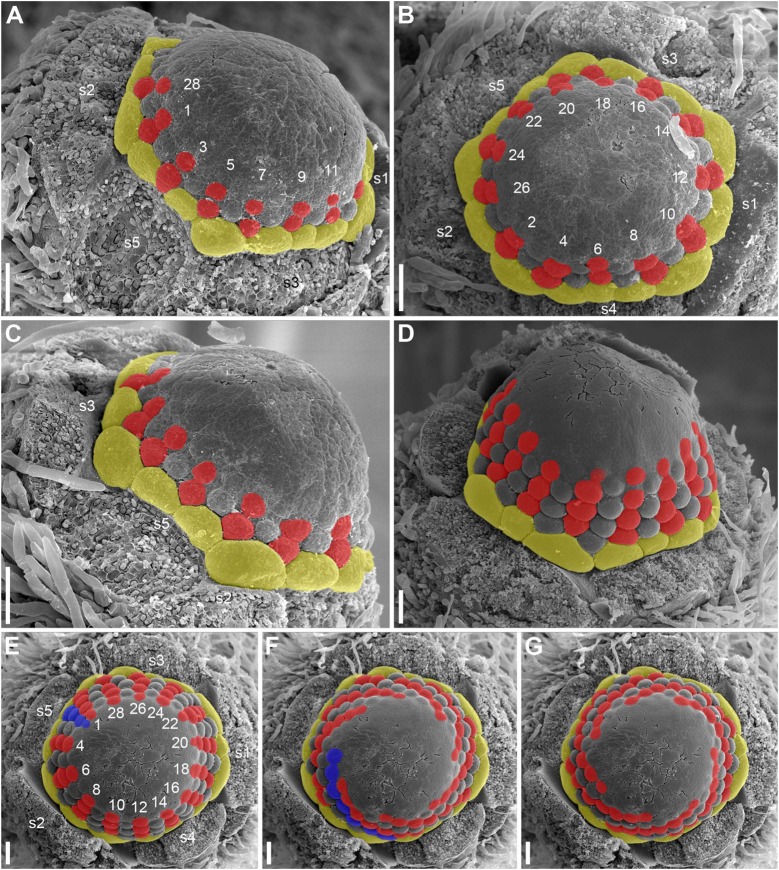
Early androecium development in *Nuphar lutea* (SEM). **(A)** Side view of flower with 15 petals and a 14.5-merous androecium whose diagram is provided in Figure 9B. Orthostichies of stamens are highlighted. Note that the first stamen of the orthostichy 3 is shifted toward the level of the petals. **(B,C)** Top and side views of flower with 14 petals and 13-merous androecium whose diagram is provided in Figure 9D. Orthostichies of stamens are highlighted. Side **(D)** and top **(E–G)** views of the flower with at least 15 petals and a 14.5-merous androecium whose diagram is provided in Figure 9E. Stamen orthostichies are highlighted in panels **(D,E)**. Two sets of stamen parastichies are highlighted in panels **(F,G)**. Yellow, petals; red (and blue), stamen orthostichies in panels **(A–E)** and stamen parastichies in panels **(F,G)**; s1–s5, petals in sequence of their initiation. Stamen orthostichies are numbered in panels **(A,B,E)** in the same way are in Figures 6B,D,E. Scale bars = 100 μm in **(A–G)**.

**FIGURE 14:**
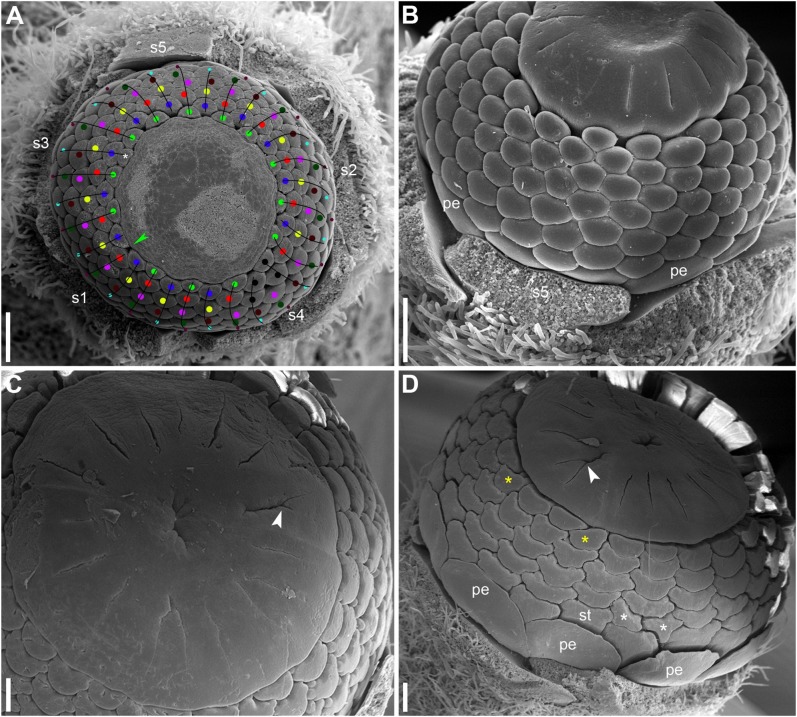
Late stages of flower development in *Nuphar lutea* (SEM). **(A)** Flower whose diagram is provided in Figure 9G. Apparent stamen whorls are indicated by dots of different colors. Black dots indicate stamens whose placement in particular whorls is problematic. Apparent orthostichies are indicated by lines. Green arrowhead indicates the position where an inner whorl stamen is missing. There is also an ‘extra’ stamen inside the innermost whorl (asterisk); note deformation of the receptacle (or very young gynoecium) in the radius of this stamen. **(B)** Flower whose diagram is provided in Figure 9H. **(C,D)** Flowers with some carpel clefts bifurcating (white arrowheads). Note the occurrence of some asymmetric stamens in panel **(D)**. Asymmetric are two stamens occupying a double position in a parastichy (white asterisks) and two stamens in the inner part of the androecium (white asterisks). Diagram of **(D)** is provided in Figure 9I. pe, petals; s1–s5, sepals; st, stamens. Scale bars = 300 μm in panels **(A–D)**.

**FIGURE 15:**
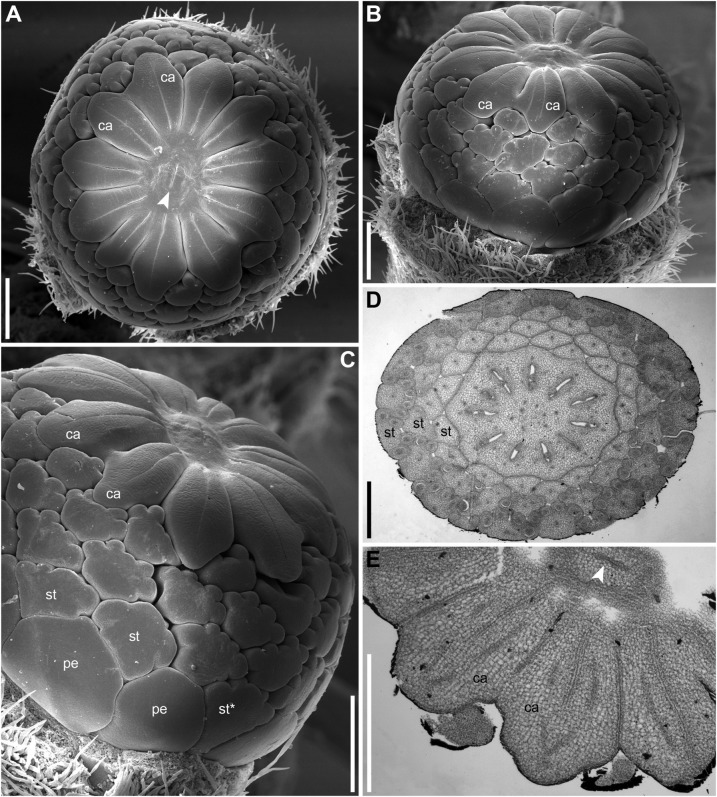
Flower of *Nuphar pumila* whose diagram is provided in Figure 10D (**A–C**, SEM; **D,E**, LM). **(A)** Top view. **(B,C)** Side views. **(D)** Cross-section at the level of the ovary. **(E)** Cross-section above the level of the ovules. ca, carpels; pe, petals, st, stamens; st*, stamen attached below the level of the other outermost stamens. Scale bars = 500 μm in panels **(A–E)**.

The petals form a series around the flower perimeter. In young flowers, their margins are not overlapping and they are all inserted at almost the same level (Figures 12, 13). At later stages, with increased width of the petals some overlapping of margins can be found (Figures 14D, 15B,C). The petals remain short and do not play important roles in protecting stamens and carpels throughout flower development.

There is a relatively long plastochron between the initiation of the last sepal and the petals. During this time the floral apex becomes more convex. The convexity increases as new organs appear and maintains until all the stamens are produced. Petals differ in shape in early as well as late developmental stages and it may be misleading to use relative size of petal primordia as indication of sequence of their initiation. Petals situated in the sectors of sepals 1, 2 and 3 (these are marked by asterisks in Figures 9, 10) initiate before petals in the sectors of the sepals 4 and 5, at least in *N. lutea* (Figures 12A–D). Petals closer to alternisepalous positions are often larger than other petals (note especially the large petal primordia between sepals 1 and 3 in Figures 13A,B). On the other hand, there are flowers in which all petals in the sectors of the sepals 1 and 2 are larger than other petals (for example, Figure 13B). The corolla is pentagonal in outline reflecting the occurrence of the five lage sepals. The three petals in front of the sepal 1 and the three petals in from of the sepal 2 are the largest ones, the three petals in front of the sepal 3 are slightly smaller and the three and two petals in the sectors of the sepals 4 and 5 are the smallest (Figure 13B). Variation of petal numbers in the five sepal sectors is summarized in Table 2.

**TABLE 2:** Variation of petal numbers in sectors of sepals 1–5 in the examined material of the two species of *Nuphar*.

	**Sector of sepal 1**	**Sector of sepal 2**	**Sector of sepal 3**	**Sector of sepal 4**	**Sector of sepal 5**
*N. lutea*	3	3–4	3–4	2–3	2–3
*N. pumila*	2–3	3	3	2	1–2

### Androecium

The first stamens initiate soon after the petals and normally occupy alternipetalous positions. As petal initiation is delayed in the sectors of the sepals 4 and 5, the very first stamen primordia can be observed in the other sectors when the last petals are yet not recognizable (black arrowheads in Figures 12B,C). The stamens form successive alternating whorls whose initiation is centripetal and rapid. These whorls, however, are not always well-recognizable due to small size of stamen primordia compared with the size of the floral apex and occasional chaotic patterns of stamen arrangement.

At the early stages of development, the petals and stamens are similar in shape and relatively small, though the stamen primordia tend to be more circular in outline. In a few cases we observed individual primordia inserted slightly above typical petals and below typical stamens. We were uncertain in identification of these primordia as future petals or stamens in young flowers (e.g., the organ marked by question mark in Figure 11C and the first member of the orthostichy 3 in Figures 9B, 13A). Later, when the thecae of anthers start to differentiate, the transition between petals and stamens is easy to determine. In our experience, the organs initiated in an intermediate position develop as stamens (see the organ labeled st^∗^ in Figure 15D).

As the outermost stamens usually alternate with petals, an ‘ideal’ flower would contain N petals, 2N of stamen orthostichies and two equal sets of N left and N right parastichies. Real androecia deviate from this ‘ideal’ scheme to a greater or lesser degree (Figures 9, 10). Due to small size of primordia in comparison with the entire floral apex, the regular whorled pattern established by petals is difficult to maintain and the phyllotaxis in most flowers becomes partly chaotic. The degree of irregularity varies from flower to flower. Some (usually 1) orthostichies can be ‘missing’ or ‘added’ in particular sites (relative to what can be expected based on the petal number) which leads to presence of unequal subsets of parastichies. Indeed, when the number of orthostichies is 2N-1, then there are sets of N and N-1 parastichies of opposite directions.

Variation in the number of parastichies can be illustrated by some examples (Figure 13). In the flower in Figure 13A (see also its diagram in Figure 9B), the orthostichies 1, 3, 5, 7, 9 are all in alternipetalous positions, but the orthostichy 11 is antepetalous. This is because one of the three petals in the sector of the sepal 3 is smaller than the other petals and there is no antepetalous orthostichy in the radius of this petal. Because of the absence of this orthostichy, the total of the orthostichies of this flower is 29 despite the presence of 15 petals (Figure 9B).

The flower in Figures 13D–G has at least 15 (possibly 16, Figure 9E) petals and 29 stamen orthostichies (Figure 13E). There are sets of 15 (Figure 13F) and 14 (Figure 13G) parastichies of opposite directions. It is easy to figure out where an expected orthostichy is missing (Figure 13D): in the left and central parts of the image, stamen orthostichies colored red are antepetalous, but they are alternipetalous in the right part of the image. The transition is in the sector of the smallest petal where an orthostichy is absent.

The flower in Figures 13B,C (diagram in 9D) has 14 petals, but only 26 stamen orthostichies. The petals in the sector of the sepal 4 are smaller than other petals and thus two expected stamen orthostiches are missing here (Figure 13B).

The number of stamens is not always equal in all orthostichies (Figures 9, 10). Alternipetalous orthostichies are sometimes one organ longer than the antipetalous ones, and this is what can be expected in a whorled flower. In some cases, just one or a few orthostichies deviate in their organ number. These deviations are mostly localized at the beginning or at the end of the orthostichies. The latter case is illustrated in Figure 14A, where there is an orthostichy that is one stamen longer than would be expected (white asterisk) and another orthostichy that is one stamen shorter than would be expected (green arrowhead). There is also a sector where precise recognizing of parastichies is problematic (black dots, Figure 14A). There are instances when some orthostichies decline or, alternatively, appear half way to gynoecium. Stamens in double positions can be sometimes observed (Figure 9F).

In general, developmental data revealed that the differences in the overall stamen number between *N. lutea* and *N. pumila* are due to the lower number of whorls as well as the lower number of orthostichies in the latter species (Figures 9, 10).

### Gynoecium

There is a long plastochron between androecium and gynoecium initiation. The gynoecium starts as a low elevation with lobes usually protruding in free areas between the stamen primordia of the final whorl (Figure 14A). The individual carpels appear simultaneously as radial slits (Figures 11D,E, 14B) located on the lobes of the floral apex (or it can be interpreted as a lobed young gynoecium). The number of carpels initiated does not always exactly correspond to the number of the innermost stamens (or to 1/2 of the number of stamen parastichies) because of intervention of chaotic patterns of stamen arrangement (Figures 9, 10). Flowers with branching slits indicating incomplete individuality of carpels are found in *N. lutea* (Figures 14C,D, white arrowheads). Similar incompletely subdivided carpels have been illustrated as early as by Trecul (1845).

In *N. lutea*, the central portion of the dome-shaped apex is not involved in carpel formation and remains undifferentiated. The carpel tips do not grow above the initial gynoecial surface. Instead, all the gynoecium enlarges as a whole by intercalary growth and carpel cavities become deeper. This developmental pattern indicates that completely ascidiate carpels form a single whorl and are congenitally united up to their tips. Only in a few flowers at the latest developmental stages, weak grooves between distal parts of the carpels were found; these never reached the margin of the stigmatic disc (not shown). The length of the carpel slits is less than 1/2 of the radius of the gynoecium, and the slits are located in peripheral parts of the radii (Figure 14B). The peripheral part of the gynoecium containing the carpel cavities elongates more extensvely than the central area. As a result, a shallow depression appears in the central area (Figure 14B). During subsequent extensive growth of the gynoecium, the upper surface of the gynoecium becomes flat and disc-shaped (Figures 14C,D). Its peripheral area grows radially and the lobes corresponding to individual carpels become much less pronounced. The central depression becomes sealed by irregular growth of more peripheral parts of the gynoecium. As a result, a number of folds can be seen in the center of the gynoecium. Their number and shape are irregular and the folds are in no way related to individual carpels. Sometimes, sealing of the depression takes a more regular form (not shown).

In *N. pumila*, the lobes of the gynoecium are initially almost as weakly pronounced as in *N. lutea* (Figures 11D,E), but with subsequent growth of the gynoecium the lobes become conspicuous and the carpel slits extend into the lobes (Figures 11F, 15A–C). There is evidence of mechanical pressure of growing lobes on adjacent stamens. The effect of pressure is only visible on late developmental stages, when anthers adjacent to gynoecium lobes are sometimes partially rotated by displacement (Figure 15C). There is no central depression (Figure 11E). While at early stages the capels are united with each other throughout their length, late in development their distalmost parts are normally free, though closely spaced. There are narrow grooves between the adjacent carpels (Figures 15A–C,E). Some of these intercarpellary grooves are incomplete and disappear in their peripheral parts (consider slits between the pairs of labeled carpels in Figures 15A–C,E).

Apart from single-whorled (Figures 11D,E), two-whorled gynoecia are found in *N. pumila*. The flower in Figure 11F is the most instructive in this respect. Here, the outher whorl of the gynoecium is distorted, because one of the innermost stamens (labeled st in Figure 11F) lies exactly of a carpel radius. The corresponding carpel is therefore shifted toward the center of the flower: the inner end of the carpel slit is closer to the center than in all other outer whorl carpels. There are three inner whorl carpels, two with short slits and one with a very short slits. The inner ends of the two short slits are at the same distance from the center of the flower as in the unusual outer whorl carpel. The entire gynoecium is rather asymmetrical (Figure 11F). Formation of single inner world carpel just in the center of another flower is documented in some flowers (Figure 15). The inner whorl carpels are sterile. On cross-sections, they can be recognized in the distal part of the gynoecium only (Figure 15E) and not at the level of the ovary (Figure 15D).

## Discussion

### Establishment and Maintainance of Shoot Chirality in Ontogeny

A remarkable feature of rhizomes of both studied species of *Nuphar* is that the direction of the ontogenetic spiral (clockwise or anticlockwise) is repeated in all rhizome branches (i.e., the shoots are homodromous; terminology: Braun, 1835; Dormer, 1965), so that all vegetative progeny of a given plant maintains shoot chirality. Along with genetic markers, this aspect can be used in population-level studies to assess relative roles of vegetative and seed reproduction.

Developmental mechanisms responsible for maintaining shoot chirality in lateral branches may be related to spatial differences between transversal anodic and transversal cathodic positions in the axil of a subtending leaf. The anodic end of a leaf is oriented in the direction up the ontogenetic spiral of phyllotaxis while the cathodic end is oriented toward the beginning of the ontogenetic spiral (Korn, 2006). The first leaf of rhizome branch is always on the anodic side of its subtending leaf in *Nuphar* (Figures 4A,B). Patterns differ among various cases of stabilized anodic/cathodic asymmetry in angiosperms (Korn, 2006). For example, in inflorescence (thyrse) of *Dioscorea tokoro*, the bracteole of the first flower of a cincinnus always lies on the cathodic side of the axil of its subtending leaf, so the pattern is reverse to what is observed in *Nuphar* (Remizowa et al., 2010a).

As soon as branching takes place on lateral sides of a rhizome in studied species of *Nuphar*, subtending leaves of the branches are always obliquely inserted. As nicely illustrated by Chassat (1962), the branch is thus somewhat displaced from the median position in the axil of its subtending leaf. But the situation is very different on the two sides of the main axis. On one side, the obliquity of leaf bases is in the direction of the ontogenetic spiral whereas on the other side of the main axis the obliquity of leaf bases is nearly perpendicular to the ontogenetic spiral. These impressive differences have no impact on the pattern of initiation of phyllotaxis in lateral branches: the first leaf of the lateral branch is still in an anodic transversal position. We believe that its position is being determined close to the apex of the main axis where the obliquity of the subtending leaf yet not manifested.

The embryo of *Nuphar lutea* has a bilateral symmetry (Meyer, 1960). In mature seed, it contains two cotyledons (organ homologies after Tillich, 1990) and a plumule with two leaves (Guttenberg and Müller-Schröder, 1958). These two leaves lie in a plane between the two cotyledons (Meyer, 1960; Haines and Lye, 1975), and the direction of ontogenetic spiral cannot be yet determined. Shoot chirality becomes pronounced only with appearance of subsequent leaves when certain leaf appears away from the intercotyledonary plane (either to the left or to the right of it). We believe that the choice between its left or right position is environmentally determined, possibly by gravitropism. Initiation of the third leaf takes place during seed germination (Guttenberg and Müller-Schröder, 1958). If the seed orientation is horizontal, the left and right positions relative to the intercotyledonary plane differ with respect to their proximilty to the Earth. It is likely that gravitropism also plays key roles in determining direction of asymmetric intercalary elongation of cotyledons in Nymphaeaceae (Sokoloff et al., 2014).

Our hypothesis on the roles of gravitropism could be tested experimentally. The experiment should include germination of a sample of seeds of *N. lutea*, with each seed being precisely fixed in a horizontal position. After emergence of epicotyledonary leaves 1, 2 and 3, their position relative to the Earth and shoot apex should be recorded in each seedling. If the leaf 3 always appears on the same side of the shoot apex (e.g., always on the lower side or always on the upper side), then its position and thus the direction of phyllotaxis is determined by gravitropism. If the results turn out to be negative, possible roles of gravitropism in pre-determining future position of the third leaf at the stage of seed development can be further tested. The experiment will require germination of seeds developed within a fruit in various orientations relative to the Earth. Finally, a hypothesis on direct genetic inheritance of shoot chirality can be tested by germination of progeny of self-pollination of plants with clockwise and anticlockwise rhizome spirals. As *N. lutea* is self-compatible (Ervik et al., 1995; Lippok and Renner, 1997), such experiments can be performed.

### Flower Arrangement in *Nuphar*

*Nuphar* and *Nymphaea* share a characteristic pattern of flower arrangement. As demonstrated long time ago (e.g., Raciborski, 1894a) and confirmed in subsequent studies (Cutter, 1957a, b, 1958; Weidlich, 1976a, b; Schneider et al., 2003; Grob et al., 2006), flowers of *Nymphaea* do not possess subtending bracts, but develop in such positions along the rhizome that the flowers can be at least superficially viewed as ‘replacing’ vegetative leaves in certain positions of ontogenetic spiral. The situation in *Nuphar* is essentially the same, with the difference that there is a tiny phyllome (or two phyllomes) at the base of the pedicel. As revealed earlier (Cutter, 1959; Moseley, 1965, 1972; Schneider et al., 2003) and supported by the present study, none of these tiny phyllomes is situated directly on the rhizome. In both genera, there is a tendency for producing flowers in positions N, N + 2 along the rhizome.

As branching is normally axillary in seed plants, a null-hypothesis that should be tested for a flower that has no obvious subtending bract is that the flower occupies a terminal position. At least for *Nuphar*, this hypothesis can be rejected using arguments summarized in the next paragraph.

If the flowers are morphologically terminal, then the rhizomes are sympodial and the continuation of the rhizome should be a lateral axis developing in the axil of the uppermost foliage leaf. Homodromous sympodial systems superficially resembling a continuous (monopodial) axis with Fibonacci pattern of phyllotaxis are well documented in a few angiosperms, for example in *Pinguicula* (Lentibulariaceae), where a lateral continuation shoot develops in the axil of the uppermost foliage leaf below a reduced 1-3-flowered umbel (Grob et al., 2007; Degtjareva and Sokoloff, 2012). In the case of *Pinguicula*, the first leaf of a continuation shoot is in an anodic position relative to the subtending leaf (like in rhizome branches of *Nuphar*). As a result of this position, only vegetative leaves of the sympodial system mimic a continuous Fibonacci spiral. The flowers (more precisely, umbels, Wydler, 1857; Degtjareva and Sokoloff, 2012) are not members of this spiral. Therefore, the similarity between the sympodial system of *Pinguicula* and the rhizome of *Nuphar* and *Nymphaea* (Raju, 1969) is only superficial (Grob et al., 2007; Degtjareva and Sokoloff, 2012). We can imagine a slightly different situation, where the first leaf of all continuation shoots is in a cathodic position and each elementary shoot is terminated in a flower. Theoretically, such a system will produce a rhizome that fits the features observed in *Nuphar*. However, (1) it is unclear why the position of the first leaf is always cathodic in the hypothetic continuation shoots and always anodic in the actually observed rhizome branches and (2) the proposed sympodial system does not allow occurrence of two flowers as neighboring members of the hypothetical ‘composite’ Fibonacci spiral in *Nuphar*. A continuation shoot should bear at least one foliage leaf before producing a terminal flower. This foliage leaf is required as a subtending leaf of the next order continuation shoot. In other words, the sympodial model nicely explains the situation of the occurrence of two flowers in positions N and N + 2 (then the leaves N-1 and N + 2 subtend continuations shoots), but the sympodial model fails to explain the occurrence of two flowers in positions N and N + 1. The latter situation is rare, but its occurrence is precisely documented in *Nuphar lutea* (Figure 2A of the present study, see also Dormer and Cutter, 1959).

Based on the evidence outlined above, we fully support earlier conclusions (Raciborski, 1894a, b; Cutter, 1957a, b, 1958, 1959; Chassat, 1962; Moseley, 1972; Schneider et al., 2003; Endress and Doyle, 2009) that the rhizome of *Nuphar* is monopodial. Next questions, which are closely related to each other and discussed in the literature cited above are (1) whether the flowers are lateral to the rhizome or to the RU axis (which is then a reduced lateral inflorescence), (2) whether a tiny basal phyllome (or any of the fwo phyllomes) is a (flower-subtending) bract or a sepal homolog and (3) whether the abaxial basal phyllome belongs to the rhizome axis and is just shifted onto its axillary branch (recaulescence) or the abaxial phyllome belongs to the lateral axis, which thus totally lacks a subtending leaf. These problems are difficult to resolve and we do not think that all the hypotheses are really testable. Moseley (1972) highlighted importance of vascular anatomy (described in Moseley, 1965) and meristem histology in resolving some of these problems. As documented by Moseley (1965), what we describe as a short common stalk of RU remarkably differs from the long flower pedicel in its vasculature. Our preliminary observations (E.S. El, unpubl. data) fully support this conclusion. The common stalk has a ring of vascular bundles, whereas the pedicel above the common stalk has two concentric rings (or stelar and cortical systems) of bundles. In the distal part of the common stalk, the bundles form a vascular complex composed of a continuous circular ring with a cross-bar, and from this there arise the concentric rings of bundles supplying the long floral pedicel (Moseley, 1965, 1972). Using anatomical sections, Moseley (1965) found a circumferential constriction just above the proximal vascular complex demarcating exteriorly the common stalk of RU. These aspects of vasculature (in form of procambial strands) are already recognizable the stage of sepal initiation, long before the intercalary elongation of the pedicel (Moseley, 1972). As pointed out by Moseley (1972), his data as well as the earlier anatomical data of Cutter (1957b, 1959) prove that the scale-like phyllome belongs to the proximal common stalk and its vasculature or procambial strand is derived from the vasculature of the common stalk. Taking into account the fact that rhizome branches and flowers appear in similar positions (forming pairs of N, N + 2), Moseley (1972: 279, see also discussion in Schneider et al., 2003: S287, S289) concluded that the short common stalk at the base of the RU is not part of the peduncle of the flower, but is, rather, ‘a short axis which is either a reduced vegetative axis (a possibility considered by Cutter, 1957b) or a vestigial inflorescence axis.’ He then favored the idea that *Nuphar* may formerly have had an inflorescence with more than one flower and concluded that the scale-like phyllome(s) do not belong(s) to the flower and should be termed bract(s). As pointed out by Moseley (1972), the meristem of the common stalk has the same histological zonation found in the rhizome apex. He uses this as evidence in favor of his interpretation of the common stalk as a reduced rhizome branch of inflorescence axis, but in our view significance of this observation should not be overestimated. Indeed, the same histological zonation is maintained until the early stage of androecium development (Moseley, 1972).

Our developmental data further support some arguments of Moseley (1972). The plastochron between the initiation of the scale-like phyllome and the first sepal is longer than between the five sepals. The sequence of sepal initiation must be very rapid as it was not possible to find any flower with just one sepal initiated. In RUs with scale-like phyllomes initiated but sepals yet absent, a floral apex can be recognized as a distinct entity. SEM allowed visualizing that the floral apex has well-defined borders throughout its circumference. It does not look like a direct continuation of the common stalk of the RU. With these developmental data, it is tempting to suggest that the scale-like phyllome belongs to the lateral axis and the flower is formed in its axil (i.e., the flower terminates a third order axis). This conclusion agrees with vascular anatomy. We do not insist that this is the only possible conclusion. Initiation of flower-subtending bract and its axillary flower by a common primordium is documented in a range of angiosperms (e.g., Remizowa et al., 2013). The (first) scale-like phyllome always occupies an abaxial position, and it is rather difficult (if not impossible) to prove or disprove the idea that it belongs to the rhizome and junited with the lateral axis (recaulescence). Recaulescence is known in many angiosperms, including some early-divergent lineages such as *Amborella* (Endress and Igersheim, 2000) and some magnoliids (Endress and Lorence, 2020). What can be inferred with more confidence is that the flower and the abaxial phyllome belong to different axes. In particular, this conclusion is supported by relative arrangement of sepals and the abaxial phyllome. Though relative arrangement of sepals is highly conserved in *Nuphar* and they can be easily numbered from sepal 1 to sepal 5 in either clockwise of anticlockwise sequence, the abaxial phyllome in most cases cannot be placed as a member of the same series preceeding the sepal 1 (Figures 4C–E). The direction of the angle between this phyllome and the sepal 1 is in most cases opposite to the direction of the angle between sepals 1 and 2 (Figures 4C,D).

Unfortunately, like earlier authors (Schneider et al., 2003), we were unable to study development of RUs with two scale-like phyllomes. None of the earlier authors provided information on relative arrangement of sepals and the second phyllome. In both flowers associated with two phyllomes studies here, the *second* phyllome could be viewed as a member of the series series of sepal arrangement preceeding the sepal 1 (Figures 4G,H).

In summary, it is most likely that the phyllome 1 is a flower-subtending bract and the phyllome 2 is a bracteole (prophyll). We prefer interpreting the phyllome 1 as belonging to the lateral axis and the flower as terminating a third order axis, but it is difficult to reject a possibility that the phyllome 1 belongs to the rhizome axis and the flower is terminating a second order axis (Raciborski, 1894a; Chassat, 1962; Endress and Doyle, 2009).

### Choice of Developmental Programs During Rhizome Development

Our study confirms earlier observations on patterns of distribution of vegetative leaves, RUs and rhizome branches along rhizomes of *Nuphar* (Raciborski, 1894a; Cutter, 1957a, b, 1958, 1959; Dormer and Cutter, 1959; Chassat, 1962). The most remarkable regularity is that RUs or a lateral branch and RU frequently form pairs in positions N, N + 2. Sometimes, this series is continued as N + 4. In *Nymphaea*, much longer series of flowers in even positions can be found. As revealed by Dormer and Cutter (1959; see also Dormer, 1965) and by the present study, given that there is RU in the position N, the probability of the occurrence of another RU in positions N + 1, N + 3 and N + 5 is very low in *Nuphar* (Figure 3C). Dormer and Cutter (1959) revealed that apart from this tendency there is a peak of probability of the occurrence of RUs at distances of 11 to 15 positions from an existing RU and interpreted this a periodicity of a longer magnitude. However, they did not analyze probabilities of the occurrence of RUs at distances longer than 21 positions in the ontogenetic spiral. Our data support the conclusion that there is an area of generally low probabilities before the distances of 11 to 15 positions. There is only a weak depression of our graph after this area (Figure 3C). We believe that this depression can be explained by secondary effect from the flowers occurring at distances of 11 to 15 positions. Thus, there are two phenomena (1) a tendency of producing flowers in even positions after an existing flower that disappears after the position N + 4 in *Nuphar* but continues further on in *Nymphaea* (Dormer and Cutter, 1959) and (2) a tendency of inhibition of flowering in certain area after an existing flower. The latter tendency is especially strong in *Nuphar advena* (Dormer and Cutter, 1959 found no RUs at all in positions 3–7). It is very difficult to imagine any environmental factors potentially responsible for these regularities. For example, no periodicity related to seasonality of growth can be seen.

While interpreting these empirical data, one needs to consider that the rhizome apex is nearly flat in waterlilies and therefore physical distances between young organs do not follow the sequence of the ontogenetic spiral. The positions N and N + 2 are closer to each other than the positions N and N + 1. We hypothesize that young primordium of RU or the site of future primordium produces a positional signal (morphogen) that in high concentrations stimulates development of subsequent primordia as RUs but in lower concentrations stimulates their development as vegetative leaves. As can be concluded from surgical experiments (Cutter, 1958), the regulation must take place before the visible appearance of organs on the rhizome apex.

The ideas proposed in the previous paragraph do not explain all features observed in rhizomes of *Nuphar*. Indeed, it is intriguing that the positions with rhizome branches follow the same regularity as those of RUs. If the branch is present in the position N, a RU develops in the position N + 2 (sometimes vice versa). Thus we hypothesize a two-step process of developmental regulation. At the first step, as outlined in the previous paragraph, positions of (pairs of) lateral axes are laid down on the rhizome apex. At the second step, if the first position of a pair is located on a lateral side of the rhizome (relative to the ground level), then a subtending leaf plus a lateral rhizome can arise here instead of RU. This step should involve environmental factors such as gravitropism. See Supplementary Data 3 for more detailed explanations of the proposed two-step regulation.

There are impressive differences between developmental programs of rhizome branches and RUs. Thus what do they have in common and what may happen at the proposed first step of the regulation (which must take place extremely early in development)? Apparently, at this step branching as such is being allowed. As highlighted by Chassat (1962) and confirmed by the present study, leaves that do not subtend branches do not have any traces of even reduced lateral buds, which is not common in angiosperms.

### Five Sepals in Two Unequal Whorls

Interpretation of the perianth organs of Nymphaeaceae and Cabombaceae as sepals and petals or tepals is disputable (Les et al., 1999; Endress, 2001; Ronse De Craene et al., 2003; Schneider et al., 2003; Padgett, 2007; Warner et al., 2009; Ronse De Craene, 2010; Coiro and Barone Lumaga, 2018). Hiepko (1965) concluded that petals of *Nuphar* and *Nymphaea* are not homologous to each other. We use the terms sepals and petals as purely technical, with no claim of petal homologies between waterlilies and eudicots (see Endress, 2006; Yoo et al., 2010; Ronse De Craene and Brockington, 2013).

Members of the section *Nuphar*, including the two species studied here, usually possess five sepals (Padgett, 2007). Sepal aestivation is quincuncial, a condition that is also known in a range of eudicots (Ronse De Craene, 2010). The sequence of sepal initiation agrees with the aestivation pattern and likely determines the latter. Spiral initiation of sepals in core eudicots is an example of spiral organ initiation in whorled flowers (Endress and Doyle, 2007). At first glance, two ideas on sepal arrangement in *Nuphar* can be proposed: (1) the calyx is spiral (spiral flowers are relatively common in basal angiosperms) and (2) the calyx is like in most core eudicots single-whorled, pentamerous. None of these ideas is confirmed. As pointed out by Endress (2001), although in *Nuphar* the outermost five or six (in the section *Astylus*, Padgett, 2007) organs appear sequentially in a spiral pattern, the position is in two whorls. This seems to be effected by a longer plastochron between the third and fourth organ of the flower (Endress, 2001). Our data apparently confirm this idea (Figures 6D, 11A). As such, delayed initiation of some sepals does not indicate that the calyx is necessarily two-whorled. For example, initiation of sepals 4 and 5 is delayed in the pentamerous calyx of some Cistaceae while in other members of the family sepals 3–5 are retarded in initiation (Nandi, 1998). Slightly unequal plastochrons were found in sepal development of some Hydrangeaceae (sometimes with a longer plastochron between sepals 3 and 4, Roels et al., 1997) and Loasaceae (Hufford, 1989).

As suggested by Endress and Doyle (2007), precise interpretation of organ arrangement as spiral or whorled should be based on analyses of divergence angles between the organs. Within a whorl, neighboring organs are equidistant, but the angle is different at the transition from one whorl to another; in spiral systems, divergence angles between successive organs along the ontogenetic spiral are more or less equal (Endress, 1987; Endress and Doyle, 2007). In a whorled pentamerous calyx, the theory predicts angles of 72° between all adjacent sepals (Figure 4I). In a trimerous calyx, angles of 120° are expected. In a calyx with five sepals in a Fibonacci spiral, angles between *adjacent* organs are unequal (this follows from equal angles of 137.5° between *successive* organs): two angles are 52.5° and three others are 85° (Figure 4J). We tested these ideas using our material of *N. lutea*. At first glance, the results do not fit any theory: mean values are about 73° for two of the five angles between adjacent sepals, 66° for two other angles and about 81° for the fifth angle (Figures 4K,L). The differences between these mean values are significant (Figure 4L). The inferred angles have nothing in common with what is predicted by the Fibonacci spiral pattern (Figure 4J). We interpret our data in the following way. There are three outer whorl sepals. Unlike the common situation, the angles between the outer whorl sepals are unequal: about 146° between sepal 1 and 2, 132° between sepals 2 and 3 and only about 81° between sepals 3 and 1. This is why only two inner whorl sepals are present. There is not enough space for the inner whorl sepal initiation in the sector between the sepals 1 and 3. The sepals 4 and 5 appear in two wider sectors between the outer whorl sepals. Importantly, our quantitative analysis revealed that the typical position of the sepal 4 is exactly between the outer whorl sepals 1 and 2 (mean angles are inferred as 72.5° and 73.6°, but these differences are not significant, Figure 4L). The sepal 5 is inserted exactly between the sepals 2 and 3 (Figure 4K). The two-whorled calyx of *Nuphar lutea* maintains the most important feature of the whorled phyllotaxis: the alternation of elements of successive whorls. Each inner whorl sepal is equidistant from the adjacent members of the outer whorl. In contrast to spiral systems, the position of the sepal 4 does not seem to depend on the position of the previously initiated sepal 3, but exclusively on the positions of the adjacent sepals 1 and 2. In the same way, the position of the sepal 5 does not depend on the sepal 4.

Special attention should be paid to use of a consistent way of scoring characters related to floral phyllotaxis in evolutionary analyses (Sauquet et al., 2017). The example of *Nuphar* shows how the ideas based on similar observations can be formulated in different ways. For example, Ronse De Craene et al. (2003) stated that *Nuphar* is occasionally pentamerous by the loss of one sepal of the inner perianth whorl (3 + 2). Endress (2004, 2006) described the perianth of *Nuphar* as spiral, apparently implying the spiral sequence of sepal initiation. Apparently, the calyx of *Nuphar* sect. *Nuphar* should not be scored in morphological data sets as either pentamerous or spiral.

We highlight the importance of the quantitative approach for analyses of organ arrangement in eudicot flowers with five sepals and quincuncial aestivation. They are expected to follow the whorled pattern (Endress and Doyle, 2007), but data on the angles between the sepals are only rarely available. It is possible that further studies will show certain heterogeneity in this group. For example, in the asterid eudicot *Fouquieria columnaris* (Fouquieriaceae, Ericales), the angles between successively initiated sepals are 137°, 137°, 155° and 132° (Schönenberger and Grenhagen, 2005), rather than all equaling 2^∗^72 = 144° (i.e., the value expected in a calyx with five equidistantly spaced sepals).

A highly important member of Nymphaeaceae for which quantitative (and developmental) data are urgently needed is *Barclaya*, which is sister to the rest of Nymphaeaceae except *Nuphar* (Les et al., 1999; Borsch et al., 2008; Taylor, 2008; Gruenstaeudl et al., 2017; He et al., 2018). The flower of *Barclaya* has four or five outermost organs usually interpreted as sepals (Tamura, 1982; Williamson and Schneider, 1994). As pointed out (but not illustrated) by Williamson and Schneider (1994), the mode and sequence of initiation of these organs is the same as described for the sepals of other Nymphaeaceae sensu stricto genera (e.g., *Nymphaea*) with the anterior sepal initiated first, followed by simultaneous initiation of the two lateral sepals, followed lastly by initiation of the posterior sepal. This description is based on flowers with four sepals. According to Tamura (1982), *Barclaya motleyi* consistently possesses five sepals with quincuncial aestivation (just as in *Nuphar* sect. *Nuphar*), though flower orientation relative to main axis is not illustrated. It is unlikely (though not impossible) that development of such calyx follows the unidirectional pattern found in *Nymphaea* and related genera. Published developmental data are not available and urgently needed. There is a useful published image of flower of *Barclaya longifolia* with five sepals. Measurements of angles between all visible organs leaves a question on its interpretation as (1) spiral or (2) whorled with 3 + 2 sepals and 3 + 2 outer petals or (3) whorled with 5 sepals + 5 petals open (Supplementary Data 4). Clearly, a quantitative approach is needed here. The first interpretation would contradict the idea of basically whorled nature of flowers in Nymphaeales (Endress, 2001; Schneider et al., 2003), the second interpretation may indicate a plesiomorphic similarity with *Nuphar*, the third interpretation would contradict the idea on the absence of stable pentamery in members of the basal angiosperm grade (Sokoloff et al., 2020).

### Calyx Symmetry and Orientation

We found certain autonomy of calyx development from surrounding structures in *Nuphar*. The occurrence of the left or right type of calyx does not depend on the direction of the ontogenetic spiral of the rhizome. This is in a strong contrast with maintainance of shoot chirality in rhizome branches. We hypothesize that the unstable direction of the calyx “spiral” is related to the fact that the sepals appear when the common stalk of the RU is already elongated (Cutter, 1957b, 1959; Moseley, 1972; present study). Also, there is no empirical evidence for sequential initiation of the sepals 1 and 2. The asymmetry only appears with the initiation of the third sepal, and the choice of its left or right position is likely random.

Earlier studies revealed that the flowers of *N. lutea* possess the sepal 3 in a nearly adaxial and sepal 4 in an abaxial position (e.g., Moseley, 1972). We found this as the most common pattern of calyx orientation in *N. lutea* (Figures 4C,D). Only this type of flower orientation was found in *N. pumila*, for which we had less material. Other, rare types were revealed in *N. lutea* (Figures 4E–H). The differences in calyx orientation did not affect sepal aestivation, which was always quincuncial. This is another evidence of autonomy of calyx development.

According to Moseley (1972), in *N. advena* and *N. variegata*, members of the section *Astylus* with 3 + 3 sepals, two outer whorl sepals are in transversal-adaxial positions and the third one is abaxial (note that the sequence of their initiation is not precisely documented). This differs from the typical condition in *N. lutea* and *N. pumila* (sect. *Nuphar*) where the two first-formed outer whorl sepals are transversal-abaxial and the third one is adaxial. Note that Raciborski (1894a), contrary to Moseley (1972), found two transversal-abaxial and the third adaxial outer whorl sepal in *N. advena*.

### Single-Whorled Interpretation of Corolla

Endress (2001) provided the most important and detailed study of early corolla development in *Nuphar*. He studied *N. advena* (with 3 + 3 sepals) and revealed that corolla development begins with initiation of six petals in double positions. Endress (2001) emphasized that flowers of various Nymphaeales share the occurrence of organs in double positions in the third whorl (six stamens in Cabombaceae, six petals in *N. advena*, eight or six petals in *Victoria* and eight petals in *Nymphaea* spp., see also Ronse De Craene and Smets, 1993). Rudall et al. (2009) suggested that the third family of the order, Hydatellaceae, may also fit this pattern, because the involucres of flowerlike reproductive units of *Trithuria occidentalis* possess two outer dimerous and the third tetramerous whorl of phyllomes.

We did not reveal petals in double positions in our material of *Nuphar*. We believe that all petals form a whorl in flowers of *N. lutea* and *N. pumila*. Thus, we support the idea that the merism of the third whorl is increased relative to previous whorls, but the increase is more extensive than just appearance of organs in double positions. The petals first appear in the sectors of the outer whorl sepals and later in the sectors of the inner whorl sepals. We suggest that differences in size of young petals not necessarily indicate the sequence of their initiation in *N. lutea*. Larger petals sometimes appear closer to the angles between adjacent sepals, especially in the angle between the sepals 1 and 3, i.e., in the position where one could hypothesize a loss of the third second whorl sepal. The single polymerous whorl of petals of *Nuphar* resembles polymerous single-whorled androecia of some (taxonomically unrelated) angiosperms (Nuraliev et al., 2010; Wanntorp et al., 2011). For example, *Polyscias waialealae* (= *Tetraplasandra waialealae*, Araliaceae) has 6–7 petals and a whorl of 28–46 stamens and some or all alternipetalous stamens are larger or much larger than the others (Nuraliev et al., 2010).

Apparently, the differences in corolla development between *N. lutea* and *N. advena* are not major. The flower of *N. advena* illustrated in Figure 9D of Endress (2001) has nine larger petals forming groups of three in the sectors of the outer sepals. Petals in the sectors of the inner whorl sepals are smaller. The flower in Figure 9C of Endress (2001), which is younger, has three petals in the sector of one of the outer whorl sepals. Petal initiation is retarded in the sectors of the inner sepals (especially in two such sectors). Our interpretation of the corolla in *Nuphar* agrees with a brief description in Wolf (1991) who investigated an unnamed species with five sepals.

It should be noted that the increase of merism in the third whorl is a common, but not universal pattern in flowers of Nymphaeales. Indeed, many members of *Nymphaea* subgen. *Hydrocallis* possess several regularly alternating tetramerous petal whorls (Wiersema, 1987). Thus three conditions can be found in Nymphaeaceae: (1) a single polymerous whorl of petals; (2) the first petal whorl isomerous to calyx, normally tetramerous, the second and subsequent whorls with organs in double positions, sometimes with irregularities; (3) a corolla with many regularly alternating tetramerous whorls. It is an open question whether this series can be read as an evolutionary scenario. Detailed developmental comparisons of various species of *Nymphaea* s.l., including measurements of relative sizes of petal primordia and floral apex are needed.

### Androecium Development

We support the idea that the androecium of *Nuphar* and other Nymphaeaceae is fundamentally whorled, with more or less pronounced irregularities (Endress, 2001; Schneider et al., 2003). Wolf (1991) performed detailed investigations of androecia of *Nymphaea alba* and a species of *Nuphar*. He found considerable diversity of androecia in *Nymphaea* (see also Ronse De Craene and Smets, 1993). In a few cases, Wolf (1991) observed androecia with numbers of left and right parastichies are typical for Fibonacci (divergence angle, α = 137.5°) or Lucas (α = 99.5°) spirals. He considered these cases as exceptions. In most examined flowers, the numbers of parastichies indicated the occurrence of more exotic types of phyllotaxis. For example, androecia with 8 + 11 parastiches of different directions (α = 132.2°) and 9 + 10 parastichies (α = 37.4°) were found. Flowers with chaotic androecia without clear parastichies were also found (Wolf, 1991). Androecia of *Nuphar* studied by Wolf (1991) were also diverse, including whorled and spiral patterns. In a flower with 15 petals (3 petals in front of each sepal), typical whorled pattern involved 30 orthostichies. This idea perfectly fits our data. An ‘ideal’ condition for *N. lutea* and *N. pumila* is the presence of equal numbers of antepetalous and alternipetalous orthostichies. Wolf (1991) revealed another situation in *Nuphar* that is close to our observations, namely with 16 + 17 + 33 parastichies (α = 21.7°). In his interpretation, which can be easily accepted, what we describe as orthostichies in Figure 13E are in fact parastichies (indeed, they are not strictly vertical), so that our flower has 14 + 15 + 29 parastichies.

We prefer describing flowers with N and N + 1 parastichies as whorled, but possessing a non-integer merism of N1/2. For example, the flower in Figures 13D–G is 14.5-merous. Such situation appears when a transition between two orthodox, integer values of flower merism is ‘frozen halfway,’ or a member of one whorl is amalgamated with an adjacent member of another whorl. Situations of this sort have been discussed and variously interpreted for some members of Caryophyllales (e.g., Ronse De Craene et al., 1998; Yurtseva and Choob, 2005; Choob and Yurtseva, 2007).

Application of the concept of non-integer merism, which can be seen as a complementary approach (Rutishauser and Sattler, 1985) to more orthodox view of such flowers as ‘simply’ spiral, is useful for plant groups with whorled flowers and unstable merism. Vislobokov et al. (2014) explored this while describing flower diversity in the monocot genus *Aspidistra* (Asparagaceae). Flowers of Asparagaceae normally possess 3 + 3 tepals and 3 + 3 stamens. Apart from this trimerous pattern, dimerous, tetramerous and pentamerous flowers are known in *Aspidistra*. In addition, flowers with uneven organ numbers are found, for example with 7 tepals and 7 stamens (or 5 tepals and 5 stamens). Interpreting such flowers as 3.5-merous (2.5-merous) is the simplest way of description (Vislobokov et al., 2014), also for any analyses of character evolution. Alternatives would be interpreting such flowers as tricyclic (considering also gynoecium), but tricyclic flowers are otherwise unknown in Asparagales or as spiral, but spiral flowers are otherwise unknown in monocots (Remizowa et al., 2010b). Similarly, if we accept the occurrence of integer as well as non-integer androecium merism in *Nuphar*, then the character ‘androecium phyllotaxis’ can be more safely scored as ‘whorled’ for this taxon for analyses of character evolution. This fits well the idea of the whorled nature of flowers in all Nymphaeales. Alternatively, we will need to score the character as polymorphic (whorled vs. spiral).

An interesting feature of whorled flowers with non-integer merism is that all ‘whorls’ are united into a continuous, very low spiral (in the case of Figure 13E with α = ca. 25°). When we consider a sector of such flower, it seems that it has alternating whorls of stamens, but if we try to trace all organs of a whorl it happens that one ‘whorl’ is a direct continuation of another ‘whorl.’ Apart from androecium of *Nuphar* and examples such as flowers of *Aspidistra*, similar patterns (called biastrepsis) are known as teratological cases in vegetative shoots of some angiosperms, gymnosperms (*Gnetum*) and pteridophytes (*Equisetum*) that normally possess a decussate or whorled phyllotaxis (e.g., De Vries, 1899; Venema, 1937; Snow, 1942; Bierhorst, 1971; Rutishauser, 1999).

Variation of floral phyllotaxis (including chaotic patterns) is well-documented in various angiosperms, especially in those with numerous floral organs (e.g., Endress and Doyle, 2007). The more numerous the floral organs (such as stamens and/or carpels) become, the smaller are their primordia with respect to the floral apex and therefore they become more prone to positional irregularities (Endress and Armstrong, 2011; Rutishauser, 2016). Zagórska-Marek (1994) revealed a great diversity of patterns of carpel arrangement in *Magnolia* flowers, including several ‘exotic’ types. Interpretation and use of these data depend on focus of discussion. Cladistic approaches require simplification of data. With currently available methods, performing large-scale studies of character evolution without such simplification is nearly impossible. Real taxa often differ in frequencies of certain character states rather than in stable alternative conditions (Meyen, 1973). Variation of floral phyllotaxis in gynoecium and androecium of *Magnolia* (mostly various spiral patterns, see also Xu and Rudall, 2006) and *Nuphar* (mostly more or less typical whorled patterns) nicely illustrates this statement. Many (if not most) other morphological characters in many taxa behave like floral phyllotaxis in *Magnolia* and *Nuphar*, and revealing certain infraspecific variation is probably a matter of sample size.

### Gynoecium Diversity in *Nuphar*

The lobed rather than entire edge of the stigmatic disc is traditionally used as a key diagnostic character of *N. pumila* (Padgett, 2007). The present study highlights a need of more detailed developmental studies of gynoecium in *N. pumila* using collections from various localities. We found remarkable features of gynoecium variation in this species. Radial grooves between distal parts of carpels are well-pronounced in *N. pumila*, though some of the grooves are incomplete and do not reach the margin of the stigmatic disc (Figure 15). In *N. lutea*, these grooves were only rarely found at the latest developmental stages and never reached the margin of the stigmatic disc. In the New World species *N. advena*, the intercarpellary grooves are absent (Igersheim and Endress, 1998). Thus, there is a variation between complete and incomplete congenital fusion between the ascidiate carpels in *Nuphar*. In *N. pumila*, this variation can be observed within an individual flower. When the fusion in incomplete, it can be classified as early congenital (Sokoloff et al., 2018b), because the intercarpellary grooves are yet absent at the earliest stages of gynoecium development.

We revealed the occasional occurrence of a second whorl of sterile carpels in *N. pumila*. This observation highlights the general phenomenon of developmental plasticity of the floral center in *Nuphar*. In *N. lutea* (our data) and *N. advena* (Igersheim and Endress, 1998; Endress, 2001), the gynoecium is somewhat concave in the center at early developmental stages, and more or less irregular grooves visible at later developmental stages (Figure 32 in Igersheim and Endress, 1998 and Figures 14C,D of the present study) merely represent by-products of sealing the central depression. As such, the sealed central depression can be conspicuous in longitudinal sections of young and mature flowers (Moseley, 1965, 1972). What we interpret as single central carpel in some flowers of *N. pumila* (Figure 15) cannot be interpreted as by-product of sealing the central depression, because it has a longitudinal slit located on a dome-shaped elevation just in the same way as in the peripheral fertile carpels. Moreover, we did not reveal any central depression in young gynoecia of *N. pumila* (Figures 11D,F). The flower interpreted here as having three sterile inner whorl carpels (Figure 11F) is even more instructive. It is younger than flowers of *N. lutea* that exhibit sealing of the central depression (Figures 14C,D). Based on the stages of carpel and stamen (no distinct microsporangia) development, the flower of *N. pumila* in Figure 11F is close to the flower of *N. lutea* in Figure 14B. Therefore, the three slits in central part of the gynoecium in Figure 11F should be better interpreted as slits of sterile inner whorl carpels. Note that they unlikely represent incipient grooves between fertile carpels, because the developmental stage is too early for appearance of these grooves and one of the three slits that we interpret as belonging to sterile carpels is markedly oblique with respect to the boundary between the closest fertile carpels (Figure 11F). Two-whorled gynoecium (or free carpels) is known from a member of Cabombaceae, *Brasenia* (Endress, 2013), and the inner whorl may comprise fewer carpels than the outer whorl (Rudall et al., 2009).

Gynoecia of most Nymphaeoideae possess a conspicuous protrusion of floral apex beyond the level of the carpels (Endress, 2013). The protrusion of the apex is usually not reported from *Nuphar* as well as from *Barclaya* (Williamson and Schneider, 1994; Les et al., 1999; Borsch et al., 2008). Moseley (1972: Figure 38) illustrated a short protusion of the apex inside a deep central depression in *Nuphar japonica*. Interestingly, there are published photographs of *Nuphar pumila* with pronounced central protrusion. In one of them, the protrusion is conspicuous and distally lobed (Bétrisey, 2018). It is tempting to propose that the lobing is due to the occurrence of the second whorl of sterile carpels. An apical protrusion is found in a range of taxa with carpels forming a polymerous whorl (or series: *Illicium*) scattered among angiosperm phylogeny (Endress, 2013). It is not surprising that the character turns to be homoplastic in Nymphaeaceae. Adding fossil record makes it potentially even more homoplastic (von Balthazar et al., 2008; Friis et al., 2009). The sporadic occurrence of the apical protrusion in *Nuphar* illustrates the Krenke’s rule (Meyen, 1973): a feature that is characteristic of a given taxon may be found as a rare condition in a related taxon.

### Internal and Mechanical Factors in Development

Ronse De Craene (2018) summarized the importance of mechanical pressures in angiosperm floral development. He highlighted differences between early-developmental and late-developmental pressures. The late-developmental pressures affect already formed organs without influencing their position at initiation (Ronse De Craene, 2018). For example, pressure marks of adjacent organs appear due to organ development in a confined space (Endress, 2008). Androecium development in *Nuphar* apparently provides examples of late developmental pressures. Very young stamens are more or less uniform, hemispherical in both species studied here (Figures 11C,D, 13). At late developmental stages, unusual shape and/or orientation of some anthers can be seen. These can be related to irregularities in stamen arrangement. For example, there is a stamen pair occupying a position where a single stamen could be expected in a whorled system in the flower illustrated in Figure 14D (white asterisks). These two stamens are of unusual shapes most likely influenced by mechanical pressures of adjacent stamens. The same flower has two asymmetric stamens in the inner part of the androecium (yellow asterisks). Here, the asymmetry is caused by pressure of an adjacent (left hand in Figure 14D) innermost stamen. The innermost stamens, in turn, are compressed by pressure of the late-developing (expanding outwards) stigmatic disc. The asymmetric nature of the pressure on the yellow asterisk stamens appears because the androecium orthostichies are curved in this part of the flower. Where the orthostichies are not curved (right hand part of Figure 14D), asymmetry of inner stamens is not manifested. Stigmatic disc is deeply lobed in *N. pumila*. Thus the late-appearing lobes have a stronger impact on the orientation of the innermost stamens. Some anthers thus may be turned to 90° (e.g., the anther between ‘ca’ and ‘ca’ in Figure 15C). The early-developmental pressures are believed to influence organ position and induce further changes, such as losses or duplications of organs (Ronse De Craene, 2018). In theory, there is no doubt that effects of this sort must take place in floral development. On the other hand, direct testing of this hypothesis is in many cases problematic. For example, in both species studied here, we found instances when a carpel was displaced inwards in the radius of a stamen occupying an unusual position. Clearly, a transference of positional information from androecium to gynoecium took place in these instances. But was this transference at the level of mechanical pressure or pre-patterning (see also Karpunina et al., 2019)?

As highlighted already by Raciborski (1894a), mechanical pressure does not play important roles in rhizome development of *Nuphar*, because young leaves and flowers are not in direct contact with each other at the rhizome apex. Our observations support this conclusion. Especially remarkable is the homodromous nature or rhizome branching that is maintained in spite of strong differences in positions of surrounding organs in branches occurring on the left and right sides of the rhizome. It must be concluded that these differences appear late in development, when branch phyllotaxis is already determined by internal factors. This idea does not necessarily imply that the internal factors are genetic in a simplistic sense. In particular, we do not insist that the occurrence of clockwise or anticlockwise phyllotaxis in rhizomes of *Nuphar* is genetically determined. Instead, we propose a testable hypothesis that it is environmentally determined early in plant ontogeny. Likewise, a possibility of direct genetic inheritance merits experimental testing.

## Conclusion

Species of *Nuphar* studied here exhibit a mosaic of strong stability and lability in their development. Stable patterns include the maintainance of shoot chirality in all rhizome branches, special positional correlation between rhizome branch and adjacent flower, whorled flowers, overall calyx structure and development with five sepals and quincuncial aestivation. Labile patterns are the distances between flowers along the rhizome length, the number of scale-like phyllomes associated with flower, calyx orientation and its left/right symmetry, petal, stamen and carpel number, relative petal size and the number of stamen orthostichies.

Intriguing tendencies in the arrangement of flowers along the rhizome in *Nuphar* and other waterlilies, especially the N, N + 2 pattern, are known since 19 Century, but we are still far from understanding mechanisms of their regulation. These days various advanced ‘omic’ (transcriptomic, proteomic, etc.) approaches could be used to compare primordia of various age and position relative to young flower on rhizome apex. With the great progress in understanding regulation of transition to flowering in *Arabidopsis*, some parallels could be found. Another promising direction is mathematical modeling of rhizome growth and organ differentiation in waterlilies.

Interpretation of floral phyllotaxis in *Nuphar* (and many other angiosperms) is problematic for two reasons: (1) infraspecific variation of patterns of arrangement of floral organs in and, importantly, (2) a possibility of use of different criteria to distinguish types of phyllotaxis. In the present paper, we made an emphasis on use of angles between organs while distinguishing whorled and spiral patterns. Another character is the order of organ initiation. A third character (Kitazawa and Fujimoto, 2018; Fujimoto and Kitazawa, 2020) is the pattern of overlapping between margins of adjacent organs (aestivation). Quincuncial aestivation normally correlates with sequential sepal initiation in angiosperms. Is this correlation always empirically demonstrated or sometimes taken as granted? In the case of *Nuphar*, we were unable to collect enough data showing sequential initiation of all five sepals. More importantly, in general, the order of initiation and aestivation are two different characters. There are instances in angiosperms when aestivation clearly does not follow the sequence of initiation, especially in corolla (e.g., Schoute, 1935; Endress, 1999). Use of different criteria may yield in different interpretation of one and the same flower. This is a problem for large scale studies like ancestral character reconstructions. This is why Sauquet et al. (2017) paid special attention to consistent method of character definition across all investigated taxa. Use of literature data for complex characters such as floral phyllotaxis may yield misleading data.

Having in mind all the problems outlined above, we still believe that our study provides further support of the idea that flowers of Nymphaeales are normally whorled (Endress, 2001; Schneider et al., 2003), though their merism and the number of whorls are unstable. Since the flower apex is large and organ primordia are relatively small, interaction of positional information from already formed organs takes place independently in different sectors of the flower. This can be seen (and documented quantitatively) already in the initiation of the two inner whorl sepals. This autonomy of various sectors of developing flowers is likely responsible for the great diversity of androecia and gynoecia in *Nuphar*.

## Data Availability Statement

All datasets generated for this study are included in the article/Supplementary Material.

## Author Contributions

EE performed morphological, anatomical, and developmental observations and took all SEM and LM images. DS designed the work and assembled all the figures of the manuscript. EE, MR, and DS interpreted the data and wrote the manuscript.

## Conflict of Interest

The authors declare that the research was conducted in the absence of any commercial or financial relationships that could be construed as a potential conflict of interest.
