# Supplementary material for: Developmental Flower and Rhizome Morphology in Nuphar (Nymphaeales): An Interplay of Chaos and Stability
Source: Front Cell Dev Biol. 2020 May 19;8:303. doi: 10.3389/fcell.2020.00303 (PMC7248231; doi:10.3389/fcell.2020.00303)
Supplement: Supplementary file 1 [file Data_Sheet_1.docx]

Supplementary Data 1

Arrangement of lateral structures in 19 examined rhizomes of *Nuphar lutea*. Each column is a rhizome. 0 = leaf without lateral branch; 1 = reproductive unit or leaf with lateral branch.

| 0 | 0 | 0 | 0 | 0 | 0 | 0 | 0 | 0 | 0 | 0 | 0 | 0 | 0 | 0 | 1 | 0 | 0 | 0 |
| --- | --- | --- | --- | --- | --- | --- | --- | --- | --- | --- | --- | --- | --- | --- | --- | --- | --- | --- |
| 0 | 0 | 0 | 0 | 0 | 0 | 0 | 0 | 0 | 0 | 0 | 0 | 0 | 0 | 0 | 0 | 0 | 0 | 0 |
| 0 | 0 | 0 | 0 | 0 | 0 | 0 | 0 | 0 | 0 | 0 | 0 | 0 | 0 | 0 | 1 | 0 | 0 | 0 |
| 0 | 0 | 0 | 0 | 0 | 0 | 0 | 0 | 0 | 0 | 0 | 0 | 0 | 1 | 0 | 0 | 0 | 0 | 0 |
| 0 | 0 | 0 | 0 | 0 | 0 | 0 | 0 | 0 | 0 | 0 | 0 | 0 | 0 | 0 | 1 | 0 | 0 | 0 |
| 0 | 0 | 0 | 0 | 0 | 0 | 0 | 0 | 0 | 0 | 0 | 0 | 0 | 1 | 1 | 0 | 0 | 0 | 0 |
| 0 | 0 | 0 | 0 | 0 | 0 | 0 | 0 | 0 | 0 | 0 | 0 | 0 | 0 | 0 | 0 | 0 | 0 | 0 |
| 0 | 0 | 0 | 1 | 1 | 0 | 1 | 0 | 0 | 0 | 0 | 0 | 1 | 0 | 1 | 0 | 0 | 0 | 0 |
| 0 | 0 | 0 | 0 | 0 | 0 | 0 | 0 | 0 | 0 | 0 | 0 | 0 | 0 | 0 | 0 | 0 | 0 | 0 |
| 0 | 0 | 0 | 1 | 1 | 0 | 0 | 0 | 0 | 0 | 0 | 0 | 1 | 0 | 0 | 0 | 0 | 0 | 0 |
| 0 | 0 | 0 | 0 | 0 | 0 | 0 | 0 | 0 | 0 | 0 | 0 | 0 | 0 | 0 | 0 | 1 | 0 | 0 |
| 0 | 0 | 0 | 0 | 0 | 0 | 0 | 0 | 0 | 0 | 0 | 1 | 0 | 0 | 0 | 0 | 0 | 0 | 0 |
| 0 | 0 | 1 | 0 | 0 | 1 | 0 | 1 | 0 | 0 | 0 | 0 | 0 | 1 | 0 | 0 | 1 | 0 | 0 |
| 0 | 0 | 0 | 0 | 0 | 0 | 0 | 0 | 0 | 0 | 0 | 1 | 0 | 0 | 0 | 0 | 0 | 0 | 0 |
| 0 | 0 | 1 | 0 | 0 | 1 | 0 | 1 | 0 | 0 | 0 | 0 | 0 | 0 | 0 | 0 | 1 | 0 | 0 |
| 0 | 1 | 0 | 0 | 0 | 0 | 0 | 0 | 0 | 0 | 0 | 0 | 0 | 0 | 0 | 0 | 0 | 0 | 0 |
| 0 | 0 | 0 | 0 | 0 | 1 | 0 | 0 | 0 | 0 | 0 | 0 | 0 | 0 | 1 | 0 | 0 | 0 | 0 |
| 0 | 1 | 0 | 0 | 0 | 0 | 0 | 0 | 0 | 0 | 0 | 1 | 0 | 0 | 0 | 0 | 0 | 0 | 0 |
| 0 | 0 |  | 0 | 0 | 0 | 0 | 0 | 0 | 0 | 0 | 0 | 0 | 0 | 1 | 0 | 0 | 0 | 0 |
| 0 | 0 |  | 0 | 1 | 0 | 0 | 0 | 0 | 0 | 0 | 0 | 0 | 0 | 0 | 0 | 0 | 0 | 0 |
| 0 | 0 |  | 1 | 0 | 0 | 0 | 0 | 0 | 0 | 0 | 0 | 0 | 0 | 0 | 0 | 0 | 0 | 0 |
| 0 |  |  | 0 | 1 | 0 | 0 | 0 | 0 | 1 | 0 | 0 | 0 | 1 | 0 | 0 | 0 | 0 | 0 |
| 0 |  |  | 1 | 0 | 0 | 0 | 0 | 0 | 0 | 0 | 0 | 0 | 0 | 0 | 0 | 0 | 0 | 1 |
| 0 |  |  | 0 | 0 | 0 | 0 | 0 | 0 | 1 | 0 | 0 | 0 | 1 | 0 | 0 | 0 | 0 | 0 |
| 0 |  |  | 0 | 0 | 0 | 0 | 0 | 0 | 0 | 1 | 0 | 0 | 0 | 0 | 0 | 0 | 0 | 0 |
| 0 |  |  | 0 | 0 | 0 | 0 | 0 | 0 | 1 | 0 | 0 | 0 | 0 | 0 | 0 | 0 | 0 | 0 |
| 0 |  |  | 0 | 0 | 0 | 0 | 0 | 0 | 0 | 1 | 0 | 0 | 0 | 0 | 0 | 0 | 0 |  |
| 0 |  |  | 0 | 0 | 0 | 0 | 0 | 0 | 0 | 0 | 0 | 0 | 0 | 0 | 0 | 1 | 0 |  |
| 0 |  |  | 0 | 0 | 0 | 0 | 0 | 0 | 0 | 0 | 0 | 0 | 0 | 0 | 0 | 0 | 0 |  |
| 0 |  |  | 0 | 0 | 0 | 0 | 0 | 0 | 0 | 0 | 0 | 0 | 0 | 0 | 0 | 1 | 0 |  |
| 0 |  |  | 0 | 0 | 0 | 0 | 0 | 0 | 0 | 0 | 0 | 0 | 0 | 0 | 0 | 0 | 0 |  |
| 0 |  |  | 0 | 0 | 0 | 0 | 0 | 0 | 0 | 0 | 0 | 0 | 1 | 0 | 1 | 0 | 0 |  |
| 0 |  |  | 0 | 0 | 0 | 0 | 0 | 0 | 0 | 0 | 1 | 0 | 0 | 1 | 0 | 0 | 0 |  |
| 0 |  |  | 0 | 0 | 0 | 0 | 1 | 0 | 0 | 0 | 0 | 0 | 1 | 0 | 1 | 0 | 0 |  |
| 0 |  |  | 0 | 0 | 0 | 0 | 0 | 0 | 1 | 0 | 1 | 0 | 0 | 1 | 0 | 0 | 0 |  |
| 0 |  |  | 0 | 0 | 0 | 1 | 1 | 1 | 0 | 0 | 0 | 0 | 0 | 0 | 0 | 0 | 0 |  |
| 1 |  |  | 0 | 0 | 0 | 0 | 0 | 0 | 1 | 0 | 0 | 0 | 0 | 0 | 0 | 0 | 0 |  |
| 0 |  |  | 1 | 0 | 0 | 0 | 0 | 0 | 0 | 0 | 0 | 0 | 0 | 0 | 0 | 0 | 0 |  |
| 1 |  |  | 0 | 0 | 0 | 0 | 0 | 0 | 0 | 0 | 0 | 0 | 0 | 0 | 1 | 0 | 0 |  |
| 0 |  |  | 0 | 1 | 0 | 0 | 0 | 0 | 0 | 0 | 0 | 0 | 0 | 0 | 0 | 0 | 0 |  |
| 0 |  |  | 0 | 0 | 0 | 0 | 0 | 0 |  | 0 | 0 | 0 | 0 | 0 | 1 | 1 | 0 |  |
| 0 |  |  |  | 0 | 0 | 0 | 0 | 0 |  | 0 | 0 | 0 | 0 | 0 | 0 | 0 | 0 |  |
| 0 |  |  |  | 0 | 0 | 0 | 0 | 0 |  | 0 | 0 | 0 | 0 | 0 | 1 | 0 | 0 |  |
| 0 |  |  |  | 0 | 0 | 0 | 0 | 0 |  | 1 | 0 | 0 | 0 | 0 | 0 | 0 | 0 |  |
| 0 |  |  |  | 0 | 1 | 0 | 1 | 0 |  | 0 | 0 | 0 | 0 | 0 | 0 | 0 | 0 |  |
| 0 |  |  |  | 1 | 0 | 0 | 0 | 0 |  | 1 | 0 | 0 | 0 | 0 | 0 | 0 | 0 |  |
| 0 |  |  |  | 0 | 1 | 0 | 1 | 0 |  | 0 | 0 | 0 | 0 | 0 |  | 0 | 0 |  |
| 0 |  |  |  | 1 | 0 | 0 | 0 | 0 |  | 0 | 0 | 0 | 1 | 0 |  | 0 | 0 |  |
| 0 |  |  |  | 0 | 0 | 0 | 1 | 0 |  | 0 | 1 | 0 | 0 | 1 |  | 0 | 0 |  |
| 0 |  |  |  | 0 | 0 | 0 | 0 | 0 |  | 0 | 0 | 0 | 1 | 0 |  | 1 | 1 |  |
| 0 |  |  |  | 0 | 0 | 0 | 0 | 0 |  | 0 | 1 | 0 | 0 | 1 |  | 0 | 0 |  |
| 0 |  |  |  |  | 0 | 0 | 0 | 0 |  | 1 | 0 | 0 | 0 | 0 |  | 1 | 1 |  |
| 0 |  |  |  |  | 0 | 0 | 0 | 0 |  | 0 | 0 | 0 | 0 | 0 |  | 0 | 0 |  |
| 0 |  |  |  |  | 0 | 1 | 0 | 0 |  | 1 | 0 | 0 | 0 | 0 |  | 0 | 0 |  |
| 0 |  |  |  |  | 0 | 0 | 0 | 0 |  | 0 | 0 | 0 | 0 | 0 |  | 0 | 0 |  |
| 0 |  |  |  |  | 0 | 0 | 0 | 0 |  | 0 | 0 | 0 | 0 | 0 |  | 0 | 0 |  |
| 0 |  |  |  |  | 0 | 0 | 0 | 1 |  | 0 | 0 | 0 | 0 | 0 |  | 0 | 0 |  |
| 0 |  |  |  |  | 0 | 0 | 0 | 0 |  | 0 | 0 | 0 | 0 | 0 |  | 0 | 0 |  |
| 0 |  |  |  |  | 0 | 0 | 0 | 1 |  | 0 | 0 | 0 | 0 | 0 |  | 0 | 0 |  |
| 0 |  |  |  |  | 0 | 1 | 0 | 0 |  | 0 | 0 | 0 | 0 | 0 |  | 0 | 0 |  |
| 0 |  |  |  |  | 0 | 0 | 0 | 0 |  | 0 | 0 | 0 | 0 | 0 |  | 0 | 0 |  |
| 0 |  |  |  |  | 1 | 1 | 0 | 0 |  | 0 | 0 | 0 | 0 | 0 |  | 0 | 0 |  |
| 0 |  |  |  |  | 0 | 0 | 0 | 0 |  | 0 | 0 | 0 | 0 | 0 |  | 0 | 0 |  |
| 0 |  |  |  |  | 1 | 0 | 1 | 0 |  | 0 | 0 | 0 | 0 | 1 |  | 0 | 0 |  |
| 0 |  |  |  |  | 0 | 0 | 0 | 0 |  | 0 | 0 | 0 | 0 | 0 |  | 0 | 0 |  |
| 0 |  |  |  |  | 0 | 0 | 0 | 0 |  | 0 | 0 | 0 | 0 | 1 |  | 0 | 0 |  |
| 0 |  |  |  |  | 0 | 0 | 0 | 0 |  | 0 | 0 | 0 | 0 | 0 |  | 1 | 0 |  |
| 0 |  |  |  |  | 0 | 0 |  | 1 |  | 1 | 0 | 0 | 0 | 0 |  | 0 | 0 |  |
| 0 |  |  |  |  | 0 | 0 |  | 0 |  | 0 | 0 | 0 | 1 | 0 |  | 0 | 0 |  |
| 0 |  |  |  |  | 0 | 0 |  | 1 |  | 1 | 0 | 0 | 0 | 0 |  | 0 | 0 |  |
| 0 |  |  |  |  | 0 | 0 |  | 0 |  | 0 | 0 | 1 | 0 | 0 |  | 0 | 0 |  |
| 0 |  |  |  |  | 0 | 0 |  | 0 |  | 0 | 0 | 0 | 0 | 0 |  | 0 | 0 |  |
| 0 |  |  |  |  | 0 | 0 |  | 0 |  | 0 | 0 | 1 | 0 | 0 |  | 1 | 0 |  |
| 0 |  |  |  |  | 0 | 0 |  | 0 |  | 0 | 0 | 0 | 0 | 0 |  | 0 | 0 |  |
| 0 |  |  |  |  | 0 | 0 |  | 0 |  | 0 | 1 | 0 | 0 | 0 |  | 1 | 0 |  |
| 0 |  |  |  |  | 0 | 0 |  | 0 |  | 0 | 0 | 0 | 0 | 0 |  | 0 | 0 |  |
| 0 |  |  |  |  | 1 | 0 |  | 0 |  | 0 | 1 |  | 0 | 1 |  | 0 | 0 |  |
| 0 |  |  |  |  | 0 | 0 |  | 0 |  | 0 | 0 |  | 0 | 0 |  | 0 | 0 |  |
| 0 |  |  |  |  | 1 | 0 |  | 0 |  | 0 | 0 |  | 0 | 0 |  | 0 | 0 |  |
| 0 |  |  |  |  | 0 | 0 |  | 0 |  | 0 | 0 |  | 0 | 0 |  | 0 | 0 |  |
| 0 |  |  |  |  | 0 | 0 |  | 0 |  | 0 | 0 |  | 1 | 0 |  | 0 | 0 |  |
| 0 |  |  |  |  | 0 | 0 |  | 0 |  | 0 | 0 |  | 1 | 0 |  | 0 | 0 |  |
| 1 |  |  |  |  |  | 1 |  | 0 |  | 0 | 0 |  | 0 | 0 |  | 0 | 0 |  |
| 0 |  |  |  |  |  | 0 |  | 0 |  | 1 | 0 |  | 0 | 0 |  | 0 | 0 |  |
| 1 |  |  |  |  |  | 0 |  | 0 |  | 0 | 0 |  | 0 | 0 |  | 0 | 0 |  |
| 0 |  |  |  |  |  | 0 |  | 0 |  | 1 | 0 |  | 0 | 0 |  | 0 | 0 |  |
| 0 |  |  |  |  |  | 0 |  | 0 |  | 0 | 0 |  | 0 | 0 |  | 0 | 1 |  |
| 0 |  |  |  |  |  | 0 |  | 0 |  | 0 | 0 |  | 0 | 0 |  | 0 | 0 |  |
| 0 |  |  |  |  |  | 0 |  | 0 |  | 0 | 0 |  | 0 | 0 |  | 1 | 1 |  |
| 0 |  |  |  |  |  | 0 |  | 0 |  | 0 | 0 |  | 0 | 0 |  | 0 | 0 |  |
| 0 |  |  |  |  |  | 0 |  | 0 |  | 0 | 0 |  |  | 0 |  | 1 | 0 |  |
| 0 |  |  |  |  |  | 0 |  | 0 |  | 0 | 0 |  |  | 0 |  | 0 | 0 |  |
| 0 |  |  |  |  |  | 0 |  | 0 |  | 0 | 0 |  |  | 0 |  | 0 | 0 |  |
| 0 |  |  |  |  |  | 0 |  | 0 |  | 0 | 1 |  |  | 0 |  | 0 | 0 |  |
| 0 |  |  |  |  |  | 0 |  | 0 |  | 0 | 0 |  |  | 0 |  | 0 | 0 |  |
| 0 |  |  |  |  |  | 0 |  | 0 |  | 0 | 1 |  |  | 0 |  | 0 | 0 |  |
| 0 |  |  |  |  |  | 1 |  | 0 |  | 1 | 0 |  |  | 0 |  | 0 | 0 |  |
| 0 |  |  |  |  |  | 0 |  | 0 |  | 0 | 0 |  |  | 0 |  | 0 | 0 |  |
| 0 |  |  |  |  |  | 1 |  | 0 |  | 1 | 0 |  |  | 0 |  | 0 | 0 |  |
| 0 |  |  |  |  |  | 0 |  | 1 |  | 0 | 0 |  |  | 0 |  | 0 | 0 |  |
| 0 |  |  |  |  |  | 0 |  | 0 |  | 0 | 0 |  |  | 0 |  | 1 | 1 |  |
| 0 |  |  |  |  |  | 0 |  | 1 |  | 0 | 0 |  |  | 0 |  | 0 | 0 |  |
| 0 |  |  |  |  |  | 0 |  | 0 |  | 1 | 0 |  |  | 0 |  | 1 | 1 |  |
| 0 |  |  |  |  |  | 0 |  | 0 |  | 0 | 0 |  |  | 0 |  | 0 | 0 |  |
| 0 |  |  |  |  |  | 0 |  | 0 |  | 1 | 0 |  |  | 0 |  | 0 | 0 |  |
| 0 |  |  |  |  |  | 0 |  |  |  | 0 | 0 |  |  | 0 |  | 0 | 0 |  |
| 0 |  |  |  |  |  | 0 |  |  |  | 0 | 0 |  |  | 0 |  | 0 |  |  |
| 0 |  |  |  |  |  | 0 |  |  |  | 0 | 0 |  |  | 0 |  | 0 |  |  |
| 0 |  |  |  |  |  | 0 |  |  |  | 0 | 0 |  |  | 0 |  | 0 |  |  |
| 0 |  |  |  |  |  | 0 |  |  |  | 0 | 0 |  |  | 0 |  | 0 |  |  |
| 0 |  |  |  |  |  | 0 |  |  |  | 0 | 0 |  |  | 0 |  | 0 |  |  |
| 0 |  |  |  |  |  | 0 |  |  |  | 0 | 0 |  |  | 0 |  | 0 |  |  |
| 0 |  |  |  |  |  | 0 |  |  |  | 0 | 0 |  |  | 0 |  | 0 |  |  |
| 0 |  |  |  |  |  | 1 |  |  |  | 0 | 0 |  |  | 0 |  | 0 |  |  |
| 1 |  |  |  |  |  | 0 |  |  |  | 0 | 0 |  |  | 0 |  | 1 |  |  |
| 0 |  |  |  |  |  | 1 |  |  |  | 0 | 0 |  |  | 0 |  | 0 |  |  |
| 1 |  |  |  |  |  | 0 |  |  |  | 0 | 0 |  |  | 1 |  | 0 |  |  |
| 0 |  |  |  |  |  | 0 |  |  |  | 1 | 0 |  |  | 0 |  | 0 |  |  |
| 0 |  |  |  |  |  | 0 |  |  |  | 0 | 0 |  |  | 1 |  | 0 |  |  |
| 0 |  |  |  |  |  |  |  |  |  | 1 | 1 |  |  | 0 |  | 0 |  |  |
|  |  |  |  |  |  |  |  |  |  | 0 | 0 |  |  | 0 |  | 0 |  |  |
|  |  |  |  |  |  |  |  |  |  | 0 | 0 |  |  | 0 |  | 0 |  |  |
|  |  |  |  |  |  |  |  |  |  | 0 | 0 |  |  |  |  | 0 |  |  |
|  |  |  |  |  |  |  |  |  |  | 0 | 0 |  |  |  |  | 0 |  |  |
|  |  |  |  |  |  |  |  |  |  | 0 | 0 |  |  |  |  | 0 |  |  |
|  |  |  |  |  |  |  |  |  |  | 0 | 0 |  |  |  |  | 1 |  |  |
|  |  |  |  |  |  |  |  |  |  | 0 | 0 |  |  |  |  | 0 |  |  |
|  |  |  |  |  |  |  |  |  |  | 0 | 0 |  |  |  |  | 1 |  |  |
|  |  |  |  |  |  |  |  |  |  | 0 | 1 |  |  |  |  | 0 |  |  |
|  |  |  |  |  |  |  |  |  |  | 0 | 0 |  |  |  |  | 0 |  |  |
|  |  |  |  |  |  |  |  |  |  | 0 | 1 |  |  |  |  | 0 |  |  |
|  |  |  |  |  |  |  |  |  |  | 1 | 0 |  |  |  |  | 0 |  |  |
|  |  |  |  |  |  |  |  |  |  | 1 | 0 |  |  |  |  | 0 |  |  |
|  |  |  |  |  |  |  |  |  |  | 0 | 0 |  |  |  |  | 0 |  |  |
|  |  |  |  |  |  |  |  |  |  | 0 |  |  |  |  |  | 0 |  |  |
|  |  |  |  |  |  |  |  |  |  | 0 |  |  |  |  |  | 0 |  |  |
|  |  |  |  |  |  |  |  |  |  |  |  |  |  |  |  | 0 |  |  |
|  |  |  |  |  |  |  |  |  |  |  |  |  |  |  |  | 0 |  |  |
|  |  |  |  |  |  |  |  |  |  |  |  |  |  |  |  | 0 |  |  |
|  |  |  |  |  |  |  |  |  |  |  |  |  |  |  |  | 0 |  |  |
|  |  |  |  |  |  |  |  |  |  |  |  |  |  |  |  | 0 |  |  |
|  |  |  |  |  |  |  |  |  |  |  |  |  |  |  |  | 1 |  |  |
|  |  |  |  |  |  |  |  |  |  |  |  |  |  |  |  | 0 |  |  |
|  |  |  |  |  |  |  |  |  |  |  |  |  |  |  |  | 1 |  |  |
|  |  |  |  |  |  |  |  |  |  |  |  |  |  |  |  | 0 |  |  |
|  |  |  |  |  |  |  |  |  |  |  |  |  |  |  |  | 0 |  |  |
|  |  |  |  |  |  |  |  |  |  |  |  |  |  |  |  | 0 |  |  |
|  |  |  |  |  |  |  |  |  |  |  |  |  |  |  |  | 0 |  |  |
|  |  |  |  |  |  |  |  |  |  |  |  |  |  |  |  | 0 |  |  |
|  |  |  |  |  |  |  |  |  |  |  |  |  |  |  |  | 0 |  |  |
|  |  |  |  |  |  |  |  |  |  |  |  |  |  |  |  | 0 |  |  |
|  |  |  |  |  |  |  |  |  |  |  |  |  |  |  |  | 0 |  |  |
|  |  |  |  |  |  |  |  |  |  |  |  |  |  |  |  | 0 |  |  |
|  |  |  |  |  |  |  |  |  |  |  |  |  |  |  |  | 0 |  |  |
|  |  |  |  |  |  |  |  |  |  |  |  |  |  |  |  | 0 |  |  |
|  |  |  |  |  |  |  |  |  |  |  |  |  |  |  |  | 0 |  |  |
|  |  |  |  |  |  |  |  |  |  |  |  |  |  |  |  | 0 |  |  |
|  |  |  |  |  |  |  |  |  |  |  |  |  |  |  |  | 1 |  |  |
|  |  |  |  |  |  |  |  |  |  |  |  |  |  |  |  | 0 |  |  |
|  |  |  |  |  |  |  |  |  |  |  |  |  |  |  |  | 1 |  |  |
|  |  |  |  |  |  |  |  |  |  |  |  |  |  |  |  | 0 |  |  |
|  |  |  |  |  |  |  |  |  |  |  |  |  |  |  |  | 0 |  |  |
|  |  |  |  |  |  |  |  |  |  |  |  |  |  |  |  | 0 |  |  |
|  |  |  |  |  |  |  |  |  |  |  |  |  |  |  |  | 0 |  |  |
|  |  |  |  |  |  |  |  |  |  |  |  |  |  |  |  | 0 |  |  |
|  |  |  |  |  |  |  |  |  |  |  |  |  |  |  |  | 0 |  |  |
|  |  |  |  |  |  |  |  |  |  |  |  |  |  |  |  | 0 |  |  |
|  |  |  |  |  |  |  |  |  |  |  |  |  |  |  |  | 0 |  |  |
|  |  |  |  |  |  |  |  |  |  |  |  |  |  |  |  | 0 |  |  |
|  |  |  |  |  |  |  |  |  |  |  |  |  |  |  |  | 0 |  |  |
|  |  |  |  |  |  |  |  |  |  |  |  |  |  |  |  | 0 |  |  |
|  |  |  |  |  |  |  |  |  |  |  |  |  |  |  |  | 0 |  |  |
|  |  |  |  |  |  |  |  |  |  |  |  |  |  |  |  | 0 |  |  |
|  |  |  |  |  |  |  |  |  |  |  |  |  |  |  |  | 0 |  |  |
|  |  |  |  |  |  |  |  |  |  |  |  |  |  |  |  | 0 |  |  |
|  |  |  |  |  |  |  |  |  |  |  |  |  |  |  |  | 0 |  |  |
|  |  |  |  |  |  |  |  |  |  |  |  |  |  |  |  | 0 |  |  |
|  |  |  |  |  |  |  |  |  |  |  |  |  |  |  |  | 0 |  |  |
|  |  |  |  |  |  |  |  |  |  |  |  |  |  |  |  | 0 |  |  |
|  |  |  |  |  |  |  |  |  |  |  |  |  |  |  |  | 0 |  |  |
|  |  |  |  |  |  |  |  |  |  |  |  |  |  |  |  | 0 |  |  |
|  |  |  |  |  |  |  |  |  |  |  |  |  |  |  |  | 1 |  |  |
|  |  |  |  |  |  |  |  |  |  |  |  |  |  |  |  | 0 |  |  |
|  |  |  |  |  |  |  |  |  |  |  |  |  |  |  |  | 1 |  |  |
|  |  |  |  |  |  |  |  |  |  |  |  |  |  |  |  | 0 |  |  |
|  |  |  |  |  |  |  |  |  |  |  |  |  |  |  |  | 0 |  |  |
|  |  |  |  |  |  |  |  |  |  |  |  |  |  |  |  | 0 |  |  |
|  |  |  |  |  |  |  |  |  |  |  |  |  |  |  |  | 0 |  |  |
|  |  |  |  |  |  |  |  |  |  |  |  |  |  |  |  | 0 |  |  |
|  |  |  |  |  |  |  |  |  |  |  |  |  |  |  |  | 0 |  |  |
|  |  |  |  |  |  |  |  |  |  |  |  |  |  |  |  | 0 |  |  |
|  |  |  |  |  |  |  |  |  |  |  |  |  |  |  |  | 0 |  |  |
|  |  |  |  |  |  |  |  |  |  |  |  |  |  |  |  | 0 |  |  |
|  |  |  |  |  |  |  |  |  |  |  |  |  |  |  |  | 0 |  |  |
|  |  |  |  |  |  |  |  |  |  |  |  |  |  |  |  | 0 |  |  |
|  |  |  |  |  |  |  |  |  |  |  |  |  |  |  |  | 0 |  |  |
|  |  |  |  |  |  |  |  |  |  |  |  |  |  |  |  | 0 |  |  |
|  |  |  |  |  |  |  |  |  |  |  |  |  |  |  |  | 0 |  |  |
|  |  |  |  |  |  |  |  |  |  |  |  |  |  |  |  | 0 |  |  |
|  |  |  |  |  |  |  |  |  |  |  |  |  |  |  |  | 1 |  |  |
|  |  |  |  |  |  |  |  |  |  |  |  |  |  |  |  | 0 |  |  |
|  |  |  |  |  |  |  |  |  |  |  |  |  |  |  |  | 0 |  |  |
|  |  |  |  |  |  |  |  |  |  |  |  |  |  |  |  | 0 |  |  |
|  |  |  |  |  |  |  |  |  |  |  |  |  |  |  |  | 0 |  |  |
|  |  |  |  |  |  |  |  |  |  |  |  |  |  |  |  | 0 |  |  |
|  |  |  |  |  |  |  |  |  |  |  |  |  |  |  |  | 0 |  |  |
|  |  |  |  |  |  |  |  |  |  |  |  |  |  |  |  | 0 |  |  |
|  |  |  |  |  |  |  |  |  |  |  |  |  |  |  |  | 0 |  |  |
|  |  |  |  |  |  |  |  |  |  |  |  |  |  |  |  | 1 |  |  |
|  |  |  |  |  |  |  |  |  |  |  |  |  |  |  |  | 0 |  |  |
|  |  |  |  |  |  |  |  |  |  |  |  |  |  |  |  | 1 |  |  |
|  |  |  |  |  |  |  |  |  |  |  |  |  |  |  |  | 0 |  |  |
|  |  |  |  |  |  |  |  |  |  |  |  |  |  |  |  | 0 |  |  |
|  |  |  |  |  |  |  |  |  |  |  |  |  |  |  |  | 0 |  |  |

Supplementary Data 2

**Angles between sepals in 23 measured flowers of *Nuphar lutea***

| Angle between the 1^st^ and the 3rd sepal | Angle between the 3^rd^ and the 5^th^ sepal | Angle between the 5^th^ and the 2^nd^ sepal | Angle between the 2^nd^ and the 4^th^ sepal | Angle between the 4^th^ and the 1^st^ sepal |
| --- | --- | --- | --- | --- |
| 82 | 69 | 70 | 80 | 59 |
| 89 | 65 | 61 | 73 | 72 |
| 78 | 66 | 64 | 66 | 86 |
| 77 | 78 | 63 | 72 | 70 |
| 88 | 62 | 57 | 66 | 87 |
| 87 | 55 | 79 | 72 | 67 |
| 87 | 58 | 72 | 73 | 70 |
| 76 | 83 | 57 | 76 | 68 |
| 67 | 58 | 75 | 82 | 78 |
| 81 | 64 | 68 | 72 | 75 |
| 73 | 73 | 78 | 78 | 58 |
| 86 | 66 | 60 | 70 | 78 |
| 93 | 70 | 63 | 66 | 68 |
| 78 | 61 | 64 | 77 | 80 |
| 74 | 75 | 59 | 78 | 74 |
| 86 | 60 | 65 | 79 | 70 |
| 92 | 67 | 60 | 77 | 64 |
| 70 | 65 | 75 | 84 | 66 |
| 80 | 73 | 50 | 72 | 85 |
| 85 | 59 | 72 | 66 | 78 |
| 79 | 71 | 85 | 58 | 67 |
| 82 | 58 | 64 | 81 | 75 |
| 92 | 63 | 58 | 74 | 73 |

Supplementary Data 3

**Speculations on possible mechanism of regulation of rhizome branching in *Nuphar*.**

We propose two steps of specification of organ types developing on rhizome apex. Both steps must take place at the level of pre-patterning, i.e. before visible primordia appear on rhizome apex.

On the first step of regulation, positions in spiral of organ arrangement where shoot branching is allowed are specified. The type of lateral branch (RU or lateral rhizome) is not specified on this first step. We hypothesize that if branching is allowed in a given position of phyllotaxis (N), then this site produces a positional signal (morphogen) that in high concentrations helps in allowing branching in subsequent positions, but in lower concentrations prevents allowing branching in subsequent positions. Concentration of this morphogen decreases with distance from the site N. As the rhizome apex is nearly flat, physical distances between young organs do not follow the sequence of the ontogenetic spiral. The positions N and N+2 are closer to each other than the positions N and N+1. Thus the concentration of the morphogen produced at site N is higher in the site N+2 than at the site N+1. This is why shoot branching (RU or rhizome branch) in *Nuphar* tend to occur in positions N and N+2. Overall patterns of distribution of the morphogen concentration should be rather complex, because factors of time should be considered (time period when the morphogen is produced by the site, speed of its transport from this site, speed of its destruction etc.) as well as production of the same morphogen at the site N+2. The latter factor is responsible for presence of RU in the position N+4. We believe that these ideas can be tested by mathematical modelling.

On the second step of regulation, a decision is being made on the type of branching, i.e., whether RU or subtending leaf together with lateral rhizome will appear in the sites specified for branching (i.e., most commonly the sites N and N+2). We postulate that another morphogen plays a role on the second step of regulation. Concentration of this morphogen is sensitive to gravitation, namely, it is higher in the physically lower portion of the shoot apex. As the rhizome grows horizontally, morphogen concentration should be unequally distributed in the apex, as illustrated in a scheme below. In the scheme below, a side view of shoot apex is shown (it is much more flat in reality than in the scheme). The two arrowheads on the dotted line indicate a distance from the apex where a decision on the type of branching is being made. We need to postulate that there is certain area of intermediate (not very high, not very low) concentration of the morphogen that stimulates development of lateral rhizomes, but only in those sites that were specified as allowed for branching as such on the first step of regulation. The area of intermediate concentration of the morphogen is shown as violet box in the scheme below. Black circles indicate various possible positions of sites of phyllotaxis. There are two strips along the rhizome where lateral rhizomes can appear, one on its left side and another on its right side. If a site pre-specified for branching does not fall into one of these strips (much more common situation), then it develops RU. If it falls into a left or right strip, then a subtending leaf plus lateral rhizome develops here. (Yet another factor should be employed to explain why lateral rhizomes tend to occur in positions N rather than N+2).

The proposed second step of regulation can be tested by experiments involving artificial re-orientation of growing rhizomes relative to the Earth to investigate potential roles of gravitropism.

The figure on the next page illustrates patterns of organ arrangement on the rhizome resulting from the proposed two-step process of regulation. These patterns fit our empirical data. In each scheme (A,B,C), a diagram of cross section of rhizome is shown. The rhizome is somewhat dorsiventrally flattened. Only positions N, N+1 and N+2 are shown. Angles between these positions (about 137º) are fixed, but the place of the position N (and thus all subsequent positions) relative to the dorsal, ventral and lateral sides of the rhizome can be viewed as random. If the position N is close to either left of right lateral side of the rhizome (C) then a subtending leaf plus lateral rhizome will form here. If not, then both positions pre-specified for branching (N and N+2) develop RUs (A, B).

Supplementary Data 4

**Measurements of angles between organs in a published image of *Barclaya longifolia* with five sepals.**

The following image is used: http://aquaria.palo-alto.ca.us/plants/n/Nymphaeaceae/Barclaya/longifolia/flower/.00-0-Copr_2015-Unknownv.jpg.meta/

All organs have been numbered sequentially, assuming a spiral sequence of their arrangement that appears to be manifested in differences in petal morphology.

A. Angles between successive outermost organs (1-5, sepals; 6-12, petals), assuming their spiral arrangement

| organ numbers | 1/2 | 2/3 | 3/4 | 4/5 | 5/6 | 6/7 | 7/8 | 8/9 | 9/10 | 10/11 | 11/12 |
| --- | --- | --- | --- | --- | --- | --- | --- | --- | --- | --- | --- |
| angles, degrees | 163 | 116 | 160 | 146 | 120 | 143 | 129 | 145 | 143 | 138 | 148 |

B. Angles between adjacent sepals (organ numbers as in A)

| organ numbers | 1/3 | 3/5 | 5/2 | 2/4 | 4/1 |
| --- | --- | --- | --- | --- | --- |
| angles, degrees | 83 | 53 | 62 | 85 | 77 |

C. Angles between adjacent petals (five outermost petals counted; organ numbers as in A)

| organ numbers | 6/8 | 8/10 | 10/7 | 7/9 | 9/6 |
| --- | --- | --- | --- | --- | --- |
| angles, degrees | 85 | 73 | 56 | 86 | 60 |
